# A path reconstruction method integrating dead-reckoning and position fixes applied to humpback whales

**DOI:** 10.1186/s40462-015-0061-6

**Published:** 2015-09-21

**Authors:** Paul J. Wensveen, Len Thomas, Patrick J. O. Miller

**Affiliations:** Sea Mammal Research Unit, Scottish Oceans Institute, University of St Andrews, St Andrews, Fife, KY16 8LB UK; Centre for Research into Ecological and Environmental Modelling, University of St Andrews, St Andrews, Fife, KY16 9LZ UK

**Keywords:** *Megaptera novaeangliae*, Marine mammal, Positioning, Fine-scale movement, State-space model, Bio-logging, Track reconstruction, Archival tag, Focal follow

## Abstract

**Background:**

Detailed information about animal location and movement is often crucial in studies of natural behaviour and how animals respond to anthropogenic activities. Dead-reckoning can be used to infer such detailed information, but without additional positional data this method results in uncertainty that grows with time. Combining dead-reckoning with new Fastloc-GPS technology should provide good opportunities for reconstructing georeferenced fine-scale tracks, and should be particularly useful for marine animals that spend most of their time under water.

We developed a computationally efficient, Bayesian state-space modelling technique to estimate humpback whale locations through time, integrating dead-reckoning using on-animal sensors with measurements of whale locations using on-animal Fastloc-GPS and visual observations. Positional observation models were based upon error measurements made during calibrations.

**Results:**

High-resolution 3-dimensional movement tracks were produced for 13 whales using a simple process model in which errors caused by water current movements, non-location sensor errors, and other dead-reckoning errors were accumulated into a combined error term. Positional uncertainty quantified by the track reconstruction model was much greater for tracks with visual positions and few or no GPS positions, indicating a strong benefit to using Fastloc-GPS for track reconstruction. Compared to tracks derived only from position fixes, the inclusion of dead-reckoning data greatly improved the level of detail in the reconstructed tracks of humpback whales. Using cross-validation, a clear improvement in the predictability of out-of-set Fastloc-GPS data was observed compared to more conventional track reconstruction methods. Fastloc-GPS observation errors during calibrations were found to vary by number of GPS satellites received and by orthogonal dimension analysed; visual observation errors varied most by distance to the whale.

**Conclusions:**

By systematically accounting for the observation errors in the position fixes, our model provides a quantitative estimate of location uncertainty that can be appropriately incorporated into analyses of animal movement. This generic method has potential application for a wide range of marine animal species and data recording systems.

**Electronic supplementary material:**

The online version of this article (doi:10.1186/s40462-015-0061-6) contains supplementary material, which is available to authorized users.

## Background

Predicting a ship’s position by projecting travel direction and speed from the previous position, a technique known as ‘dead-reckoning’, has been used for centuries [1] and is the basis for modern inertial navigation systems in vehicles [2]. Since its introduction in animal bio-logging over 25 years ago [3, 4], dead-reckoning has become an established method for reconstructing fine-scale movement tracks, in particular for air-breathing marine animals that spend most of their time under water, out of sight of global positioning system (GPS) signals [5, 6].

Dead-reckoning has led to novel insights into the natural foraging and orientation behaviour of marine animals including pinnipeds (e.g. [7–10]), turtles [11], diving birds [12, 13], and cetaceans (e.g. [14–22]), and has provided important information about the behavioural responses of cetaceans to noise [23–28]. Although animals can also be localised under water using active and passive sonar (e.g. [29–33]), such techniques require transmission and/or reception of sound which is difficult to accomplish at a high resolution, and may impact the environment of acoustically-sensitive marine mammals.

Dead-reckoning for marine animals was enabled by the development of miniature animal-attached data loggers that record movement parameters such as compass heading, speed, and body orientation [34–38]. Because each dead-reckoned position depends upon the previous one, the spatial error in the track generally grows with time due to an accumulation of sensor errors, movements of water currents, and violations of the assumptions that the animal only moves through the water in the caudo-rostral direction and that buoyancy and lift forces are negligible [6]. A common source of uncertainty in dead-reckoning tracks (sometimes called ‘pseudo tracks’) is the speed of the animal. Speed may be estimated if direct measurements are missing [24], but can also be measured with a speed sensor [13] or approximated based on pitch and change in depth [39], acoustic flow noise [40], or overall dynamic body acceleration [41].

Fixes of known positions on the earth’s surface can be used to adaptively calibrate dead-reckoning sensors or to directly correct dead-reckoned positions [2]. Position fixes of marine animals are obtained, for example, by visual observation (which can be aided by the use of laser range finders and animal-attached very high frequency (VHF) transmitters) [42, 43], acoustic localisation [44, 45], light intensity-based geolocation [46], or GPS satellite telemetry. Since conventional GPS is generally not feasible for marine animals because of a long (~10-30 s) time-to-fix and high current consumption [47], new snapshot GPS technologies such as Fastloc-GPS [48–50] have quickly become popular because of their ability to acquire data sufficient to estimate location during short surface intervals [51]. Such approaches store GPS pseudorange data, which can be converted into positions after the logger is retrieved or after transmission through Argos [52] or mobile phone networks [53]. The average spatial accuracy for positions observed with Fastloc-GPS (<100 m) is much greater than for positions from Argos (0.5-10 km) or light-based geolocation (1-4°) [54–56]; therefore, the integration of Fastloc-GPS and dead-reckoning data has the potential to result in highly precise georeferenced movement tracks [57].

Most studies to date have assumed a constant bias in velocity between position fixes, essentially stretching the track to match the fixes [5] or have iteratively approximated a constant bias [24]. We describe here a new method for referencing dead-reckoning tracks to position fixes based upon state-space models (SSMs). SSMs are an appropriate statistical tool for this application because they explicitly separate the observation processes from the underlying movement process [58] and are a standard technique in integrated navigation systems for avian, automotive and naval applications [2]. In animal ecology, SSMs for track reconstruction and smoothing have been implemented as Kalman filters (e.g. [59–63]), particle filters [64], and using Markov chain Monte Marlo (MCMC) (e.g. [65–68]). Movement data of relatively low temporal resolution (e.g. collected via Argos, GPS and light-based geolocation) have been the focus of most research on marine animals, although Kalman filters have also been applied to high-resolution dead-reckoning data in combination with depth [17, 34] and depth and acoustic localisation data [69].

The rapid technological developments in bio-logging will likely result in an increasing demand for analysis methods for high-resolution data that are easy to implement and fast to compute. We achieve this in the current study by using the fine-scale dead-reckoning track to provide the expected 2-dimensional displacement in a discrete-time correlated random walk SSM that operates at the irregular but discrete temporal scale of the low-resolution positional fixes. This gives us the advantage of using the high-resolution information without the computational cost associated with running a SSM at very fine temporal scale. The disadvantage is that the uncertainty associated with the dead-reckoning track is ignored, so that our estimates of uncertainty in location at times between position fixes are underestimates. The size of the underestimation depends largely on the time between position fixes, so the method will work better for animals that make frequent surfacings.

Our study was motivated by the need for detailed whale tracks in a series of controlled exposure experiments (CEEs; [70]) on humpback whales (*Megaptera novaeangliae*) in 2011 and 2012 in waters off Bear Island and Svalbard [71–73]. These experiments were aimed at quantifying the behavioural effects of 1.3-2 kHz naval active sonar and to test the effectiveness of a mitigation measure called ‘ramp up’ [74]. The whales were tagged with multi-sensor data loggers and Fastloc-GPS loggers, and were subsequently tracked by visual observers from a small boat. The distance between the whale and the sound source during experiments was a crucial parameter; therefore, the main objective of this study was to develop SSMs to reconstruct whale tracks from dead-reckoning, Fastloc-GPS, and visual observations. A secondary objective was to quantify the spatial accuracy of the Fastloc-GPS and visual (range and bearing) observations in dedicated tests, so that the observation errors included in our models would be realistic. The track reconstruction method presented here is easy to implement and has potential application for a wide range of marine animal species and data recording systems. Example software and model code that users can adapt for their own research questions are provided as supplementary materials (Additional file 1).

## Methods

### Study subjects, equipment and data collection

Thirteen humpback whales were tagged with multi-sensor digital recording tags (DTAGs, v2; [6]) with a Fastloc-GPS logger (F2G 134A, Sirtrack, New Zealand) mounted on top, at northern latitudes between 74.00° and 79.03° and eastern longitudes between 9.79° and 20.68° in 2011 and 2012 (Table 1). The tags were attached to each whale with suction cups using a pneumatic tag launching system (ARTS; [72]) or using a 15-m carbon fibre pole, cantilevered in a bow-mounted oarlock [75]. The DTAGs had 1 or 2 hydrophones and recorded sound with 16-bit resolution, at 96 kHz sampling rate. The DTAGs also recorded 50 Hz pressure, temperature, tri-axial acceleration and tri-axial magnetic field-strength data. Prior to tag deployment, the internal clock of the DTAG was set to local time (synced to 1 s) using a GPS receiver. Fastloc-GPS loggers were configured to record a GPS snapshot almost instantaneously after the device emerged from the water during a surfacing of the whale. The minimum time interval between GPS snapshots was set to 30 s.

**Table 1:** Summary of the data sets

Whale	DTAG ID	FGPS ID	Initial position		Track duration	Position fixes		Model runtime
			Latitude	Longitude		Visual	FGPS	
			°N	°E	h	#	#	h
1	mn11_157a	29 420	75.141	14.603	14.7	105	451	22.5
2	mn11_158a	29 409	74.832	16.715	7.6	70	20	0.8
3	mn11_160a	29 409	74.651	15.236	13.0	116	0	1.0
4	mn11_165e	29 409	78.074	11.824	11.3	123	205	7.6
5	mn11_176b	-	77.563	12.537	2.9	44	-	0.2
6	mn12_161a	29 420, 29 510	77.556	11.277	10.9	186	0	2.6
7	mn12_164a	29 409	77.798	10.073	7.7	122	391	17.1
8	mn12_164b	29 409	77.824	9.793	3.8	68	100	2.3
9	mn12_170b	29 409	77.512	11.633	8.3	87	249	21.6
10	mn12_171b	29 409, 29 510	79.032	10.612	7.8	127	646	78.4
11	mn12_178a	29 420	74.867	17.767	7.6	50	159	4.2
12	mn12_179a	29 420	74.051	20.675	8.5	75	202	6.0
13	mn12_180b	29 409, 29 420	73.993	20.398	7.6	115	730	54.7

For each humpback whale are given the IDs of the DTAG and Fastloc-GPS loggers, geographical coordinates of the initial observed position, track duration, number of position fixes obtained by visual observation and Fastloc-GPS, and computational runtime of the model. The DTAG ID contains information about the species, year, Julian day and tag-of-day; for example, ‘mn11_157a’ refers to the first tag (‘a’) deployed on a humpback whale (*Megaptera novaeangliae*) on day 157 of 2011

Focal follows of tagged humpback whales were conducted from an 8-m long water jet propulsion boat with an elevated observer platform. Each tag contained a very high frequency (VHF) radio beacon which aided tracking of tagged whales. The observers on the platform measured the angle to the whale relative to the boat’s heading using a protractor at the time of the animal’s first surfacing observed at least 2 min after the previous sighting was recorded. Simultaneously, the (radial) line-of-sight distance to the whale was measured using a laser range finder (LRF), or estimated by eye. Because the eye height was only ~3 m, we assumed that the difference between the line-of-sight distance and the distance over the earth’s surface [76] was negligible. To aid locating the whale at the surface, angles-of-arrival of the VHF signals from the tag were made visible to observers by a digital radio direction finder system (DFHorten, ASJ Electronic Design, Horten, Norway) connected to four 4-element Yagi antennas. All visual tracking information (e.g. range, bearing, coordinated universal time (UTC), range estimation method, and GPS positions of the observation boat at 1-s intervals) were stored in a MS Access database via the software Logger (International Fund for Animal Welfare, Yarmouth Port, MA); the data collection protocol is described in more detail elsewhere ([71]).

### Dedicated accuracy tests

#### Fastloc-GPS

We conducted ‘dry’ tests with Fastloc-GPS loggers in 2011 and 2012 at four sites (56.33°N, 2.78°W; 69.68°N, 18.99°E; 78.24°N, 15.54°E; 64.92°N, 23.25°W) to quantify the spatial accuracy of each data logger. Measurements were collected with the same three loggers (device IDs: 29 409, 29 420, and 29 510) that were deployed on humpback whales. During the calibration tests, the three devices were in a stationary position, spaced >25 cm apart, and recorded GPS snapshots every 30 s in an outdoor space with an open view of the sky. We used manufacturer-provided software (Archival USB, v1.11, PathTrack, UK) to offload the pseudoranges and convert them into position estimates based upon the relevant daily broadcast satellite ephemeris data. Information stored for each spatial location included the UTC time stamp, number of GPS satellites used in the position calculation, and the residual value of the position solution.

For error calculations, we assumed that the true position of a logger was equal to the median of all of the observations for each logger. The geographical coordinates of the observations were converted into Universal Transverse Mercator (UTM) coordinates so that positional errors (the difference with the median coordinate) could be expressed in meters. An observation was excluded from analysis if the residual value of its position solution was >30 (no unit); this threshold was recommended by Sirtrack ([77]) and adopted by other studies using Fastloc-GPS (e.g. [78–80]). The error measurements were divided into bins based upon the number of satellites (‘#satellite bins’) from which data were recorded (4, 5, 6, 7, 8, and 9–12). Scaled *t* distributions were fitted using maximum likelihood estimation via the ‘MASS’ package (v7.3-19, [81]) in the software R (v3.0.2, [82]) to estimate the parameters of the observation error distributions for each #satellite bin and each orthogonal dimension. The goodness-of-fit of the distributions were checked with one-sample Kolmogorov-Smirnov tests.

#### Visual tracking

Five tests were conducted in June 2012 in waters near Tromsø, Norway (69.79°N, 19.19°E) and waters near Longyearbyen, Svalbard (78.56°N, 14.95°E) to quantify the accuracy of visual observations. The observers estimated range (radial distance) and bearing to an orange heavy duty inflatable buoy that had a diameter of 1.2 m. A handheld GPS receiver (Etrex Legend HCx, Garmin, Schaffhausen, Switzerland) with EGNOS capability was attached on top of the buoy for recording its GPS positions for groundtruthing. A total of seven observers participated in the tests (the same individuals who conducted the focal follows on tagged humpback whales); two or three observers participated at the same time. The observation boat from where visual estimates were made sailed an undetermined course, making occasional turns, matching operations during whale tracking. To imitate the data coverage during real focal follows, the boat was within <200 m from the buoy for roughly 50 % of the estimates but occasionally moved to distances of around 1 km. One person (the ‘data recorder’) stored the estimates in the software Logger and gave vocal commands. Once every 2 min, the data recorder called out “Ready”, which indicated to the observers to start looking for the target and to the driver to adopt a steady course. About 10 s later, the data recorder called out “Mark”, which indicated to the observers to make their estimates and write them down on paper. We limited the time that the observers could look at the target because this influences the accuracy of the range estimates [83]. The estimates for range were made visually by the observers, and protractors were used to measure the bearing relative to the heading of the boat. The same observation boat and data collection protocol were used during the focal follows of the humpback whales (details in [71]).

The absolute bearing (relative to true north) to the whale from the boat at the time of a sighting was calculated by adding the boat’s course-over-ground derived from GPS to the relative bearing to the whale. Linear errors in range and bearing were calculated as the difference between the visual estimates and the ‘true values’ derived from the GPS positions of the buoy and the observation boat. The linear range errors were clearly a function of range itself (and thus ‘heteroskedastic’), so percent error in range was used instead of absolute error (i.e. a multiplicative error model was used). To test for potential remaining range-dependency, we fitted a linear regression model to the percent error in range as function of true range in MATLAB (v8.1; The Mathworks, Natick, MA). A wrapped Cauchy distribution was fitted to the angular errors in bearing in R using the package ‘circular’ (v0.4-7, [84]).

### Process model

Position fixes (with respect to the Earth frame of reference) of the humpback whale at the sea surface naturally occurred at irregular time intervals. The process model in our model framework operated on the relatively coarse time scale of these fixes. This greatly reduced computational time, but had the disadvantage that the dead-reckoning errors were not fully incorporated and thus underestimated the positional uncertainty between fixes. The SSM described here is therefore an approximation to a full SSM that would run on the finer time scale of the tag data. The humpback whale data set contained relatively high rates of position fixes (average of 0.1-1.9 observations/min; n = 13; Table 1), and at those rates the contribution of dead-reckoning on the uncertainty was relatively minor compared to the uncertainty from the positional observations. We therefore combined a fairly simple process model with relatively realistic positional observation models.

For the process model, we defined *J* as the number of position fixes, *j* = 1,…,*J* as the index over these fixes, and Δ_*j*_ as the time interval between *t*_*j*_ and *t*_*j* + 1_. We wrote scalars in italic and vectors in bold italic. Only the horizontal (*xy*) plane was considered because the depth of the whale (i.e. the *z*-coordinate of its position) was measured with a highly accurate sensor and therefore assumed to be observed without error. The process model essentially combined the whale’s position given by the high-resolution dead-reckoning track (see next section) with a velocity correction term. Specifically, given an initial unobserved whale position ***x***_1_, the unobserved whale positions ***x***_*j*_ at *t*_*j*_ were derived using the algorithm

1 $$ {\boldsymbol{x}}_{j+1}={\boldsymbol{x}}_j+{\boldsymbol{d}}_j^{dr}+{\boldsymbol{v}}_j^{cor}{\Delta}_j, $$

where ***d***_*j*_^*dr*^ is the whale’s expected displacement over Δ_*j*_ given by the uncorrected dead-reckoning track, and ***v***_*j*_^*cor*^ is the velocity correction for the track segment. This correction term can be interpreted as the mean ‘bias’ or ‘drift’ in velocity over Δ_*j*_ [1, 5], although in many studies using movement models these qualifications refer to the mean velocity of the animal itself [85]. To reflect our belief that ***v***^*cor*^ could only change slowly over time, we assumed that its process was a non-directional first-order Gaussian random walk,

2 $$ {\boldsymbol{v}}_{j+1}^{cor}\sim MVN\left({\boldsymbol{v}}_j^{cor},\boldsymbol{\varSigma} {\Delta}_j\right) $$

where the process noise variance-covariance matrix $$ \boldsymbol{\varSigma} =\left[\begin{array}{cc}\hfill {\sigma}_x^2\hfill & \hfill 0\hfill \\ {}\hfill 0\hfill & \hfill {\sigma}_y^2\hfill \end{array}\right] $$ and *σ*_*x*_^2^ and *σ*_*y*_^2^ represent the variances for the *x*- and *y*-dimension. The covariance term was set to 0 as the process noise was assumed to be independent between the two spatial dimensions. A linear relationship of ***Σ*** with Δ_*j*_ was incorporated to account for the dead-reckoning errors that grow with time.

### Determining the dead-reckoning track

We describe here how the uncorrected dead-reckoning track was derived from the high-resolution observations. As mentioned earlier, no observation models were incorporated for these tag-derived data. We defined *I* as the number of high-resolution observations, *i* = 1,…,*I* as the index over these observations, and Δ_*i*_ as the time interval between *t*_*i*_ and *t*_*i* + 1_. The whale’s uncorrected velocity ***v***_*i*_ for Δ_*i*_ was

3 $$ {\boldsymbol{v}}_i={s}_i \cos \left({p}_i\right)\left[\begin{array}{c}\hfill \cos \left({h}_i\right)\hfill \\ {}\hfill \sin \left({h}_i\right)\hfill \end{array}\right], $$

where *s*_*i*_ is the whale’s speed-through-water, and pitch *p*_*i*_ and heading *h*_*i*_ describe the orientation of the whale’s body with reference to the Earth frame [35]. Vector ***v***_*i*_ may be used to calculate the uncorrected dead-reckoning track using the algorithm ***x***_*i* + 1_ = ***x***_*i*_ + ***v***_*i*_*Δ*_*i*_; however, because the process model operated on the coarser, irregular time scale *t*_*j*_ determined by the position fixes, we integrated ***v***_*i*_ with respect to time in the domain *t*_*i*_ = [*t*_*j*_, *t*_*j* + 1_) to find the whale’s uncorrected displacement ***d***_*j*_^*dr*^ that was used in Eq. 1:

4 $$ {\boldsymbol{d}}_j^{dr}={\displaystyle {\sum}_{t_i={t}_j}^{t_{j+1}}}\left({\boldsymbol{v}}_i{\Delta}_i\right). $$

### Positional observation models

A set of equations stochastically related each whale’s unobserved position ***x***_*j*_ at time *t*_*j*_ to the observations of range (radial distance), bearing, and/or Fastloc-GPS. The observation error structures were based upon the results of the dedicated accuracy tests (see ‘Results’). Specifically, the observation model relating the observed Fastloc-GPS position, *X*_*x*,*j*_^*F*^, to the unobserved whale position for the *x*-dimension was

5 $$ {X}_{x,j}^F\sim t\left({x}_{x,j},{\sigma}_{x,q}^F,{\upsilon}_{x,q}^F\right), $$

with a similar formulation for the *y*-dimension. Parameter ***σ***_*q*_^*F*^ represents the scale and ***υ***_*q*_^*F*^ the shape (or, degrees of freedom) of the scaled *t* distribution. Because Fastloc-GPS accuracy is related to #satellites [50, 55], we used the parameter estimates obtained from the dry test data as fixed values for ***σ***_*q*_^*F*^ and ***υ***_*q*_^*F*^ (where quality *q* = 1,…,6 indexes the 4, 5, 6, 7, 8, and >8 satellite bins, respectively) in an approach similar to the use of Argos quality classes in other studies (e.g. [65]).

The observation model implemented for range between observer and whale at the surface was

6 $$ {R}_j\sim N\left({r}_j,\ {r}_j{\sigma}_m^r/100\right), $$

where *R*_*j*_ is the observed range and *r*_*j*_ is the unobserved range. Thus, we assumed that the observation error was normally-distributed around 0 %, which was close to the truth according to the visual observer tests (see ‘Results’). Scale parameter *σ*_*m*_^*r*^ represents the percent error SD for *m* = 1, 2, where range estimation method *m* = 1 if estimates were made visually (by eye), and *m* = 2 if a laser range finder was used to make the measurement. Its value for *m* = 1 was based upon the visual accuracy tests and for *m* = 2 was assumed to be 10 %. The observation model implemented for absolute bearing between the observer and the whale was

7 $$ {\varPhi}_j\sim wC\left({\varphi}_j,\ \rho \right), $$

where *Φ*_*j*_ is the observed bearing, *φ*_*j*_ the unobserved bearing, and *ρ* is the scale (or, concentration) of the wrapped Cauchy distribution that was derived from the visual accuracy tests.

Finally, we related the unobserved difference in position between the observation boat and the whale (***d***_*j*_^*bw*^ = ***x***_*j*_ − ***x***_*j*_^*b*^) to the unobserved range and bearing via a Cartesian-to-polar coordinate transformation:

8 $$ {r}_j=\left\Vert {\boldsymbol{d}}_j^{bw}\right\Vert,\ \mathrm{and} $$

9 $$ {\varphi}_j={ \tan}^{-1}\left({d}_{x,j}^{bw}/{d}_{y,j}^{bw}\right), $$

where tan^− 1^ is the four-quadrant arctangent to realise *φ*_*j*_ =(−180°, 180°]. The position of the observation boat ***x***_*j*_^*b*^ was measured with a GPS receiver with an average error of <3 m (unpublished data). This GPS receiver was located within 1 m from the visual observers; therefore, ***x***_*j*_^*b*^ was set to be equal to the Cartesian coordinates of the measured GPS positions (the model can be easily adapted to include error on the observer boat’s position).

### Data processing and model fitting

#### Pre-processing

Procedures for offload, calculation and filtering of data collected by the deployed Fastloc-GPS loggers were the same as for test data (see for details: ‘Methods – Dedicated accuracy tests’). Using a conversion from geographical to UTM coordinates, all positions of the whale and the observation boat were placed in a Cartesian coordinate system with at the origin (*x* = 0, *y* = 0) the first observed position of the whale (Table 1). We temporally aligned the position fixes of the same surfacing to further reduce computational costs. This was accomplished by 1) identifying pairs of Fastloc-GPS observations that were observed within 5 s of one another and replacing the timestamp of the last fix with that of the first (only for whales that had two GPS loggers attached), and 2) replacing the timestamps of the visual observations that were made ±5 s from a Fastloc-GPS observation by the timestamp of the Fastloc-GPS observation. The 5-s interval was judged to be the longest time interval that could not result in observations from separate whale surfacings being falsely aligned, and was based upon an exploratory analysis in which the times of position fixes were plotted on the corresponding dive profile.

For each tag record, data on depth, acceleration and magnetic field strength from the DTAG were downsampled to 1 Hz resolution (*Δ*_*i*_ = 1 s) using a DC accurate decimating filter. The whale’s pitch (*p*_*i*_) and heading (*h*_*i*_) were derived from the acceleration and magnetic field measurements following the techniques detailed elsewhere [35]. Estimates of the whale’s speed-through-water (*s*_*i*_) based upon depth rate per second divided by the sine of pitch during steep (i.e. |*p*_*i*_| > 50°) descents and ascents [39] were regressed against the uncalibrated (1-s root-mean square) noise level (*L*_*i*_) in the 66–94 Hz frequency band [21] using the model:

10 $$ \log \left({s}_i\right)\sim N\left({\beta}_0+{\beta}_1{L}_i,{\sigma}^L\right), $$

where *β*_0_, *β*_1_ and *σ*^*L*^ are model parameters. This function should be an appropriate model according to the physics of flow noise [86], although empirical verification is recommended on a case-by-case basis. Both body pitch and noise level were low-pass filtered using a zero-group-delay fast impulse response filter with a 0.15 Hz cut-off frequency to remove fine-scale temporal variations such as from fluke strokes to generate thrust [87]. The fitted function was used to predict *s*_*i*_ from *L*_*i*_ throughout the entire tag record, including the regions of shallower pitch [40, 88]. Flow noise is likely to be influenced by noise generated by the sea surface when the whale is at shallow depth; therefore, speed-through-water estimates for each period where the whale was at <5 m depth were replaced using a linear interpolation of the start and end values of the period.

#### Fitting the track reconstruction model

Model fitting was performed using Markov chain Monte Carlo (MCMC) algorithms in the software JAGS (v3.4.0, [89]) through an interface with MATLAB. We assigned uniform priors to most parameters: *σ*_*x*_ ~ *Unif*(0, 0.1), *σ*_*y*_ ~ *Unif*(0, 0.1), *v*_*x*,1_^*cor*^ ~ *Unif*(−1, 1) and *v*_*y*,1_^*cor*^ ~ *Unif*(−1, 1); only the initial position of the whale had informative priors that reflected the accuracy of its observation (Table 2). Thirteen models were fitted to the data set; one for each whale record. To assess whether parameters converged to stationary distributions, we ran two MCMC chains with different initial values. Each chain had a burn-in period of 200,000 samples and a total run length of 280,000 samples, and was downsampled (thinned) by a factor of 5 to reduce memory load. Mixing and stationarity were assessed by visual examination of trace plots and using the Brooks-Gelman-Rubin statistic $\widehat{R}$ [90]. MCMC chains were run in parallel on multiple cores of a desktop computer (Intel i7-4930 K processor with six physical cores; 32 GB of RAM; 64-bit MS Windows 7 operating system); up to three models were fitted at the same time.

**Table 2:** Prior probability distributions for all parameters estimated

Parameter	Description	Prior
*σ*_*x*_	Process error standard deviation, *x*-dimension	*Unif*(0, 0.1)
*σ*_*y*_	Process error standard deviation, *y*-dimension	*Unif*(0, 0.1)
*v*_*x*,1_^*cor*^	Initial velocity correction, *x*-dimension	*Unif*(−1, 1)
*v*_*y*,1_^*cor*^	Initial velocity correction, *y*-dimension	*Unif*(−1, 1)
*x*_*x*,1_	Initial whale position, *x*-dimension (1)	*t*(0, *σ*_*x*,*q*_^*F*^, *υ*_*x*,*q*_^*F*^)
	Initial whale position, *x*-dimension (2)	*N*(0, *R*_1_ *σ*_*m*_^*r*^/100)
*x*_*y*,1_	Initial whale position, *y*-dimension (1)	*t*(0, *σ*_*y*,*q*_^*F*^, *υ*_*y*,*q*_^*F*^)
	Initial whale position, *y*-dimension (2)	*N*(0, *R*_1_ *σ*_*m*_^*r*^/100)

Uniform priors were assumed for ***σ*** and ***v***_1_^*cor*^. Prior distributions for the initial unobserved whale position ***x***_1_ reflected our prior knowledge about the accuracy of the initial observed position (at coordinates *x* = 0, *y* = 0). These priors therefore depended upon whether the position was observed (1) using Fastloc-GPS or (2) visually. Values for the priors on ***σ*** and ***v***_1_^*cor*^ are in metres per second

#### Post-processing

The JAGS output included the posterior estimates of the low-resolution track (***x***_*j*_; the whale positions at the times of the position fixes); posterior estimates of the high-resolution track (***x***_*i*_) were calculated in a post-processing analysis. To obtain the final (corrected) position estimates with uncertainty, 3,200 high-resolution track realisations (or ‘posterior sample tracks’) were calculated from 1,600 computed iterations (10 % of the total) using the whale’s uncorrected velocity ***v***_*i*_ derived with Eq. 3, the posterior samples of the whale’s initial position ***x***_1_, and the posterior samples of the velocity correction ***v***_*j*_^*cor*^. The JAGS code of the model, an example data set, and code for data processing in MATLAB is given in Additional file 1.

### Cross-validation to assess model performance

A form of 10-fold cross-validation [91] was conducted to compare the performance of our method to other track reconstruction methods. Specifically, the cross-validation analyses tested how well out-of-set Fastloc-GPS positions were predicted by the state-space model and the other methods. Only Fastloc-GPS position fixes were part of this analysis as they were generally more accurate than the visual position fixes (see ‘Results’) and less likely to include temporal autocorrelation. First, we left out every 10^th^ Fastloc-GPS observation (the ‘validation data’) and fitted the state-space model to the remaining observations (the ‘training data’). For each observation in the validation set, we then measured the positional (cross-validation) error relative to the following horizontal track types: 1) the mean posterior track based on the state-space model fitted to the training data, 2) a track with linear interpolation between the training data, 3) a track with linear interpolation between visual position fixes (excluding fixes that occurred during the same surfacings as the validation data), and ‘forced-point’ dead-reckoning tracks that were stretched to match the training data [5] and initially calculated with 4) constant speed or 5) speed derived from flow noise. The procedure was iterated 10 times per whale, each time changing the validation set indices to leave out a different 10 % of the Fastloc-GPS observations. Cross-validation analyses were conducted for three different whales (IDs 1, 7, and 11) and positional errors were averaged within their #satellite bin to assess overall model performance.

Because the rate of Fastloc-GPS fixes was relatively high for these three whales (~1 fix every 2 min; Table 1), a second type of cross-validation was conducted in which the validation set was created by taking a series of five consecutive positions instead of a single position, leaving the next five consecutive positions in the training data set. Therefore, instead of omitting 10 % of the observations at each iteration, 50 % of the observations were omitted (periods that averaged 10 min) at each iteration, and the same Fastloc-GPS positions were part of the validation set five times. Calculation of the positional cross-validation errors was the same as described above, except that visual position fixes were excluded during the whole time interval spanning the five consecutive Fastloc-GPS observations.

## Results

### Fastloc-GPS accuracy tests

**Fig. 1:**
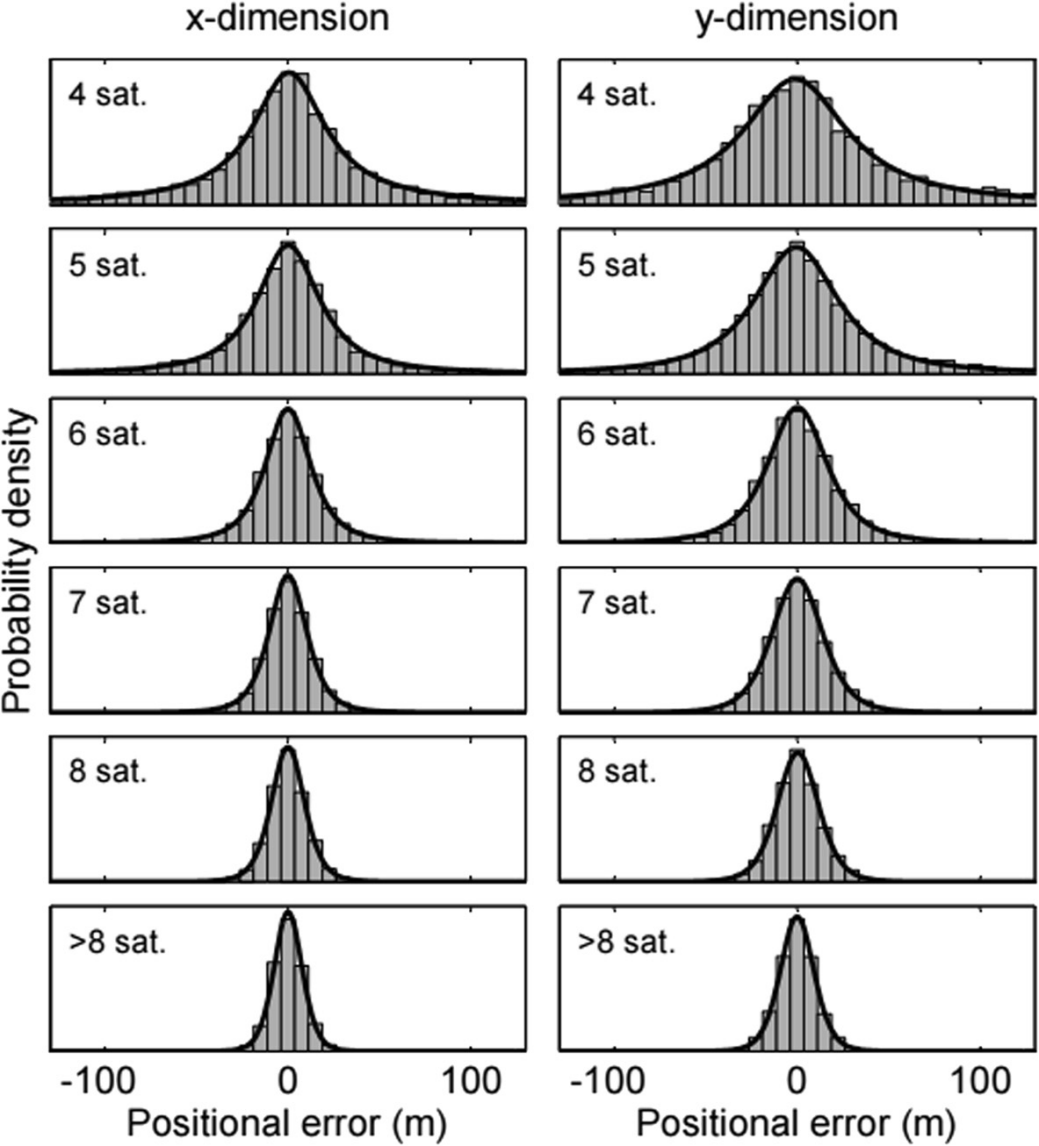
Error distributions from Fastloc-GPS accuracy tests. Scaled histograms (grey bins) of the Fastloc-GPS positional errors and the corresponding pdfs (black lines) of the scaled *t* distributions are shown as functions of spatial dimensions *x* and *y* and the number of satellites used in the position calculation. All graphs are truncated at ±130 m for clarity, although positional errors of several kilometres were occasionally observed

A total of 35,347 location observations were collected during ‘dry’ tests with Fastloc-GPS loggers (n = 3) in fixed positions, which amounted to a total of 4.9 days’ worth of data. The number of observations assigned to the 4, 5, 6, 7, 8, >8 satellite bins was 3,864 (11 %), 4,690 (13 %), 5,648 (16 %), 6,402 (18 %), 6,102 (17 %) and 8,641 (24 %), respectively. Only 0.2 % of these observations had residual values >30 and were omitted from the final data set (all sites and devices combined). The spatial errors of the three loggers were similar within each #satellite bin, although one logger (ID 29 420) acquired data from a greater number of satellites on average (7.7) than the other two loggers (6.5 and 6.7) (Additional file 2: Figure S1) and thus recorded more positions of higher accuracy. There were some indications that the errors differed somewhat across test sites, possibly because of differential weather conditions, but this comparison was limited by low numbers of observations in some of the subsets (Additional file 2: Figure S1). For both spatial dimensions (*x* and *y*), the accuracy of the Fastloc-GPS observations was positively related to the #satellites used in the position calculation (Fig. 1). The positional errors in the final data set were well described by the scaled *t* distribution (Fig. 1; Kolmogorov-Smirnov tests, p > 0.05 for each distribution). The maximum likelihood estimates and standard errors (SEs) for ***μ***^*F*^, ***σ***^*F*^, and ***υ***^*F*^ are provided in Table 3. The obtained error distributions were symmetric (*μ*^*F*^ close to 0 m) and ~1.3 times narrower in the *x*-direction than in the *y*-direction (*σ*_*y*_^*F*^/ *σ*_*x*_^*F*^; see also Fig. 1 and Additional file 2: Figure S1). Estimates for ***υ***^*F*^ increased with the #satellites from about one (Cauchy errors) for 4 satellites to about eight (approximating Gaussian errors) for >8 satellites.

**Table 3:** Fastloc-GPS test results

Parameter	Number of satellites	*X*-dimension		*Y*-dimension	
		Estimate	SE	Estimate	SE
***μ*** ^*F*^(m)	4	0.57	0.56	−1.06	0.76
	5	0.21	0.38	−0.41	0.49
	6	0.02	0.22	0.17	0.28
	7	0.01	0.16	0.10	0.20
	8	−0.01	0.14	0.39	0.17
	>8	0.08	0.09	−0.01	0.11
***σ*** ^*F*^(m)	4	24.51	0.68	34.07	0.03
	5	19.11	0.42	25.37	0.04
	6	13.10	0.23	17.12	0.10
	7	10.69	0.16	14.23	0.19
	8	9.28	0.14	11.56	0.42
	>8	7.77	0.10	9.35	0.65
***υ*** ^*F*^(−)	4	0.93	0.90	1.08	0.03
	5	1.44	0.55	1.64	0.05
	6	2.53	0.29	2.73	0.11
	7	3.91	0.21	5.32	0.34
	8	5.83	0.18	6.86	0.58
	>8	8.17	0.12	7.72	0.61

Scaled *t* distributions were fitted to the positional errors measured during the Fastloc-GPS accuracy tests. Maximum likelihood estimates and standard error (SEs) are provided for location ***μ*** ^*F*^, scale ***σ*** ^*F*^, and shape ***υ*** ^*F*^ for each spatial dimension and number of satellites used for the position calculation (#satellite bin)

### Visual accuracy tests

**Fig. 2:**
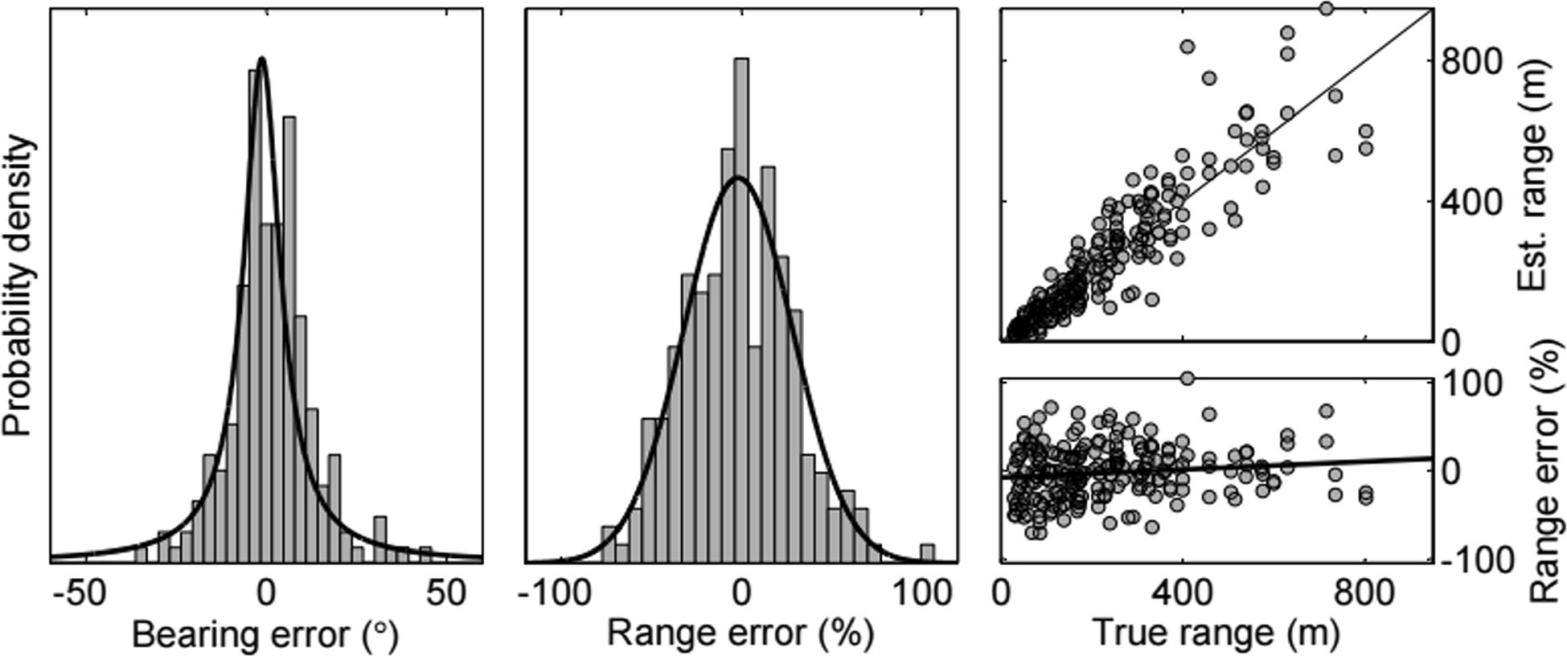
Error distributions from visual accuracy tests. Distributions of (left) angular errors in the bearing from the observer to the whale, and (middle) errors in range expressed as a percentage of true range. Grey bins represent scaled histograms of the observation errors and black lines represent the pdfs of the fitted distributions (wrapped Cauchy for bearing; Normal for range). The scatterplots in the right panels illustrate: (top) the range estimated by the observers during tests as function of the true range derived from GPS positions, and (bottom) the range percent errors vs. true range, with the fitted linear regression line indicating little tendency for under- or overestimation

The accuracy tests with human observers (n = 7) produced a total of 220 visual observations of range and bearing used to estimate location. Each test took ~40 min; the combined duration of the data collection periods was 3.2 h. Despite modest sample sizes, the percent errors in range and angular errors in bearing were reasonably well described by the Normal and wrapped Cauchy distributions, respectively (Fig. 2; Kolmogorov-Smirnov test with range data, p > 0.05). The slope of the percent error in range regressed against the true range was significantly different from 0 at p = 0.02, indicating that the percent error overestimated at close range and underestimated at large range, but this effect was very small (0.027 % per metre; Fig. 2). There was very little consistent negative bias in the estimates of range (*μ*: −2.95 %) and bearing (*μ*: −1.24°). Visual estimates of range were relatively inaccurate (*σ*_1_^*r*^: 30.2 %) compared to the bearing estimates (*ρ*: 0.897; circular SD: 11.6°). The positional uncertainty of a whale location obtained through visual observation will therefore be highly asymmetrical in Cartesian coordinates, further justifying the use of a range-and-bearing observation model to incorporate the anisotropic errors.

### High-resolution tracks of humpback whales

Visual examination of the trace plots of the estimated parameters confirmed that convergence was always reached within the burn-in phase, MCMC chains were stationary, and sufficient posterior samples were obtained. This was corroborated by $\widehat{R}$ values of ≤1.05 for each parameter (Additional file 3: Table S1). The model runtime varied greatly across whales (range: 0.2 to 78.4 h; Table 1) and depended strongly upon the number of position fixes (especially from Fastloc-GPS).

**Fig. 3:**
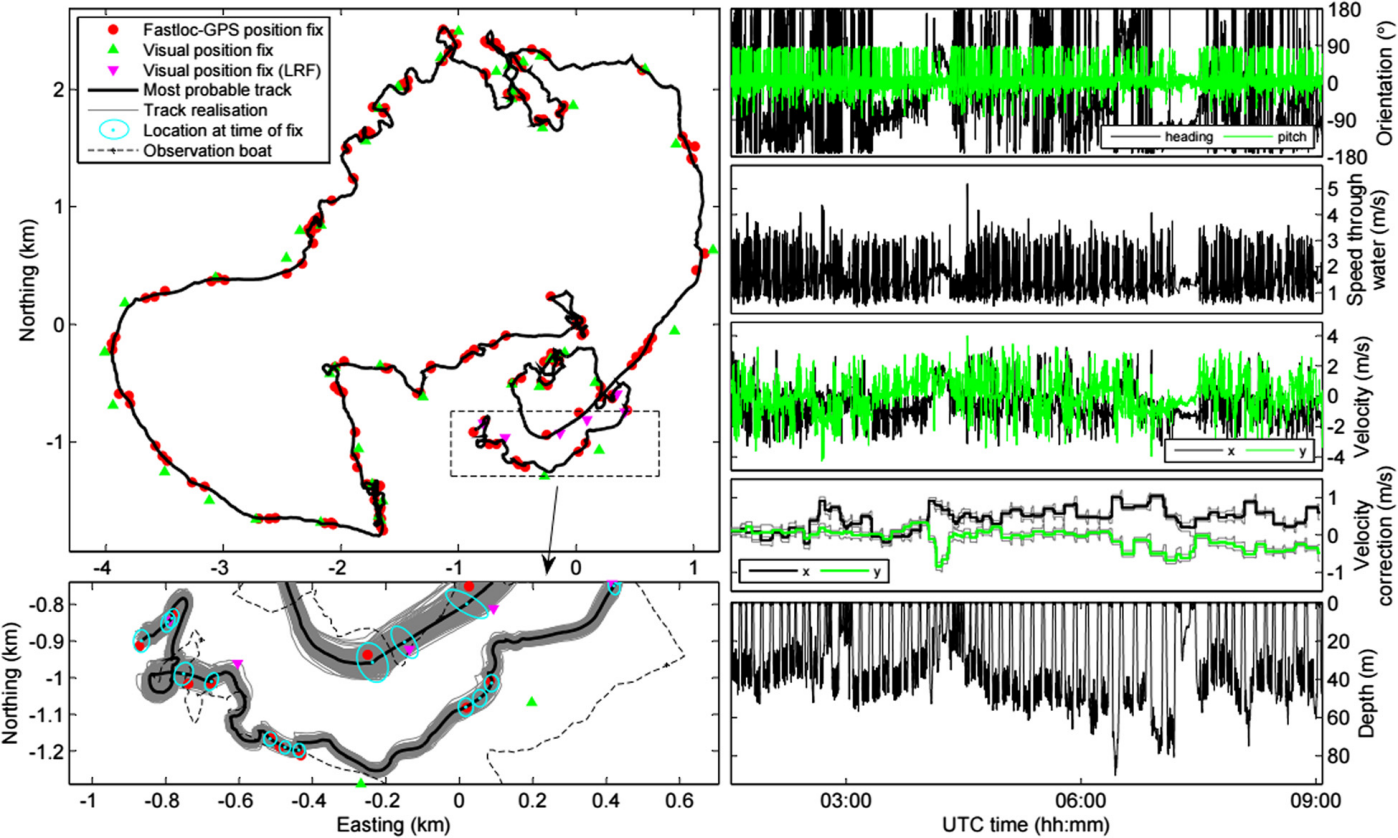
Example of a reconstructed track. Shown on the left are (top) the full, most probable track (i.e. the posterior means of *x*) and position fixes of humpback whale 11 and (bottom) a detailed view of sections of the track. Visual position fixes were derived from ranges that were estimated by eye or measured using a laser range finder (LRF). Information only shown in the bottom panel: the GPS positions of the observation boat, 10 % of the computed whale track realisations, and the most probable whale positions at the times of the fixes (***t***_***j***_) with their 95 % confidence ellipses [109]. Movement parameters of the track are shown in the panels on the right: (from top to bottom) the whale’s body pitch and heading angles measured in the Earth frame, the whale’s speed-through-water derived from flow noise, the uncorrected velocity of the whale, the posterior mean velocity correction with 95 % credibility intervals (CIs), and the depth of the whale (*z*-axis coordinate of its position)

We first provide an example of a reconstructed fine-scale track using the results for whale 11. This whale remained in an area of about 5 × 4 km (*x* × *y*) for the full 7.8 h duration of the track (Fig. 3). The whale’s horizontal movements ranged from very directional with slow clockwise turns and little short-term heading variation to very non-directional with large short-term heading variation. In general (and as expected), the most probable (posterior mean) whale positions were very close to the Fastloc-GPS fixes, further from position fixes made with laser range finder, and the furthest from position fixes for which range was estimated by eye (Fig. 3). Repetitions of bursts of speed (up to 3–4 ms^−1^) concordant with rapid changes in depth suggested that this whale performed multiple feeding ‘lunges’ (i.e. feeding events in which the animal speeds up to engulf large volumes of water and filter prey; [87, 88]) in the bottom phase of most dives. The whale’s uncorrected velocity ***v*** over the whole track ranged between −3.6 and 3.2 ms^−1^ in the *x*-direction (min/max *v*_*x*_^*cor*^: −0.2/1.0 ms^−1^) and between −4.3 and 4.0 ms^−1^ in the *y*-direction (min/max *v*_*y*_^*cor*^: −0.9/0.3 ms^−1^). Some sudden changes in ***v***^*cor*^ appeared to correspond with changes in the movement parameter values for this animal (e.g. the shallow diving period starting at 04:00 UTC). The velocity correction process for this whale was relatively volatile (posterior means for *σ*_*x*_ and *σ*_*y*_ of 0.014 and 0.012 ms^−1^, respectively) compared to that of other whales (Table 4; Additional file 4: Figures S2-S14).

**Table 4:** Posterior probability distributions

Whale	Estimated parameter					
	*σ*_*x*_ (ms^− 1^)			*σ*_*y*_ (ms^− 1^)		
	mean	SD	95 % CI	mean	SD	95 % CI
1	0.006	0.001	0.004-0.008	0.005	0.001	0.004-0.006
2	0.010	0.002	0.007-0.014	0.009	0.001	0.006-0.011
3	0.009	0.001	0.007-0.012	0.010	0.001	0.008-0.012
4	0.007	0.001	0.005-0.009	0.007	0.001	0.005-0.009
5	0.008	0.003	0.004-0.015	0.015	0.004	0.009-0.023
6	0.006	0.001	0.004-0.007	0.007	0.001	0.005-0.009
7	0.007	0.001	0.006-0.009	0.006	0.001	0.005-0.008
8	0.006	0.001	0.004-0.008	0.007	0.001	0.005-0.011
9	0.004	0.000	0.003-0.004	0.003	0.000	0.003-0.004
10	0.014	0.001	0.012-0.015	0.012	0.001	0.010-0.013
11	0.014	0.001	0.012-0.017	0.012	0.001	0.010-0.015
12	0.005	0.001	0.004-0.007	0.012	0.002	0.009-0.015
13	0.010	0.001	0.008-0.011	0.009	0.001	0.007-0.010

Mean, standard deviation (SD) and 95 % credibility interval (CI) of the marginal posterior distributions are provided for the velocity correction process SDs, *σ*_*x*_ and *σ*_*y*_. For the posterior summaries of ***v***_1_^*cor*^ and ***x***_1_, readers are referred to Additional file 3: Table S1

The complete data set of 13 whales contained large differences in movement patterns and behaviour (Additional file 4: Figures S2-S14), and detailed visual inspection of the tracks suggested that the track reconstruction model performed satisfactory under a wide range of conditions. The positional uncertainty in tracks with none or few Fastloc-GPS fixes (e.g. whales 2 and 3; Table 1) was generally greater than for tracks with many Fastloc-GPS fixes (e.g. whales 7 and 13). Clear differences in the posterior mean estimates of ***v***^*cor*^ were observed among animals (Additional file 4: Figures S2-S14); while in some cases its values remained close to 0 ms^−1^ for the entire track duration (e.g. whales 1 and 9), in others its values gradually changed over time (e.g. whale 13) or values indicated a strong consistent bias in one direction (whale 3). This between-animal variation in ***v***^*cor*^ was also reflected in the posterior mean estimates of ***σ***, which ranged between 0.003 and 0.015 ms^−1^ and were often similar between *x*- and *y-*dimensions (Table 4; Additional file 5: Figure S15).

### Model performance

**Fig. 4:**
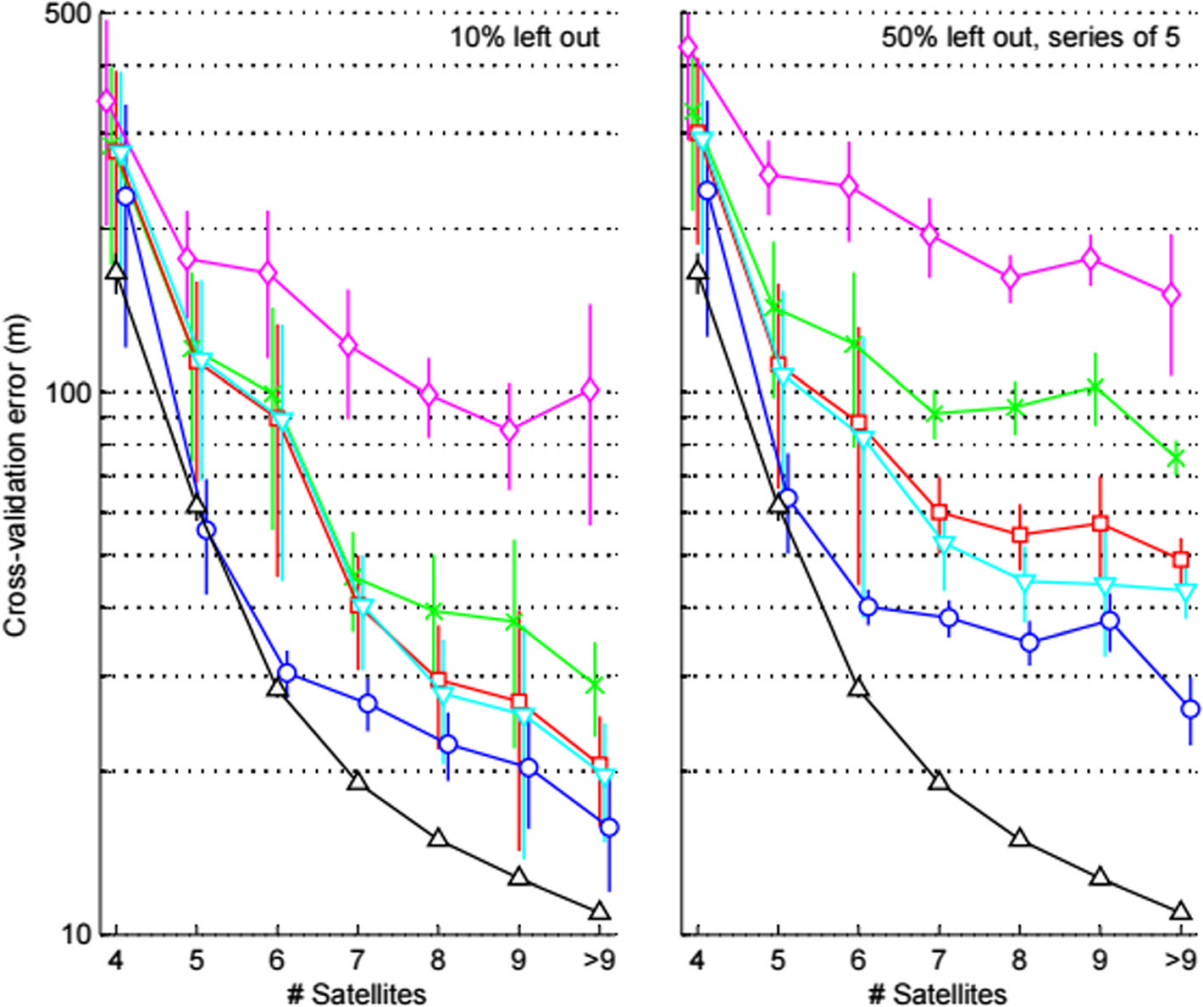
Results of the cross-validation analysis. Cross-validation errors (mean ± 2 s.e.m.) are shown as function of the number of satellites of the validation set (i.e. the out-of-set Fastloc-GPS data) for analyses where (left) single positions were omitted (10 % of data) and (right) series of five consecutive positions were omitted (50 % of data). Positional cross-validation errors were calculated for five different track types: 1) a track with linear interpolation between visual position fixes (♦), 2) a track with linear interpolation between Fastloc-GPS position fixes (**×**), ‘forced-point’ dead-reckoning tracks initially calculated with 3) constant speed (■) or 4) speed from flow noise (▼), and 5) the mean posterior track of the Bayesian state-space model (●). One-dimensional positional errors for Fastloc-GPS derived from the large data set collected during dry tests (▲) are shown for comparison (see also Additional file 6: Figure S16). Symbol horizontal positions have been offset for clarity

Results of the cross-validations were based upon a combined (n = 3 whales) validation set of 206, 247, 212, 161, 96, 44, and 29 unique Fastloc-GPS positions (for 4, 5, 6, 7, 8, 9, and >9 satellites, respectively). Positional cross-validation errors indicated that the mean posterior tracks of the Bayesian SSMs most closely approximated the validation data and the mean measurement errors from the dry tests compared to other track reconstruction methods (Fig. 4). Performance varied across methods, with the forced-point dead-reckoning tracks being, on average, more accurate than the tracks with linear interpolation between Fastloc-GPS fixes and tracks with linear interpolation between visual fixes (Fig. 4). Mean cross-validation errors decreased with increasing #satellites for all track types, indicating that the measurement errors of the validation data formed part of the cross-validation errors. As expected, the cross-validation errors were greater and the differences between methods greater when the validation sets contained blocks of 5 consecutively observations (simulating periods of ~10 min without data collection) instead of single observations (Fig. 4). However, the above results regarding which method performed best and the decreasing error with #satellites were the same for both 10 % and 50 % data removal.

## Discussion and conclusions

Accurate tracking of marine animals (e.g. mammals, penguins, and turtles) with high-resolution multi-sensor data loggers has become increasingly important in ecology and conservation biology [6, 92]. These data loggers have already provided valuable information on topics such as foraging behaviour [20, 88, 93–95], time and energy budgets [96, 97] and human impacts [26, 98, 99], but the number of methods available for analysis of marine animal movements from high-resolution data is still very limited. To partially address this gap, our study describes an effective SSM framework that is designed for relatively fast reconstruction of fine-scale tracks combining visual, Fastloc-GPS, and dead-reckoning data. Empirical data from accuracy tests formed the basis of the observation models.

Visual observation is a method that is often used for accurate tracking of marine mammals at the surface (e.g. using land-based theodolite tracking [23], boat-based focal follows [43], or stereo photogrammetry [100]), but a quantitative assessment of its accuracy, as presented in this study, is relatively uncommon. The visual accuracy tests with a floating buoy showed that the errors in range generally contributed most to the combined positional error from range and bearing observations, which is consistent with results from more extensive testing during transect line surveys [101]. The average range estimation error (SD: 30 %) was similar to those of naturalists on whale-watching vessels (25 %) and less similar to range estimates of captains (19 %) and members of the general public (45 %) on these same vessels [102].

The use of the normally-distributed percent error for range was a practical way to scale the error with distance, although a minor range-dependent effect in the transformed data remained. Error models for range based upon distributions such as the gamma or log-normal may be more appropriate in certain situations [103]. The accuracy tests were designed to emulate the real focal follows as much as possible by, for example, using the same platform and observers, and limiting the duration that the target was visible to the observers [83]. However, these tests were not exhaustive and the estimated errors were likely only reasonable approximations to the actual errors during focal follows. We did not account for observer-specific differences in the visual estimates for a number of reasons (i.e. recording who made each observation was not part of the field protocol, low sample size per observer for accuracy tests, and the estimation error of one observer from 2011 was not quantified), but future studies could incorporate observer-specific range and bearing errors within the model framework.

The estimated accuracy of the three Fastloc-GPS loggers was roughly comparable to other reports [48, 50, 55] when we quantified accuracy in terms of 1-dimensional spatial error (Additional file 6: Figure S16). For example, we found that 50 and 95 % of the errors in positions based on 4 GPS satellites were within 50 and 633 m, respectively. In comparison, the values for these respective percentiles in [48] were 50 and 810 m and in [50] were 36 and 724 m. The differences in accuracy compared to these other studies were likely caused by factors related to satellite coverage, atmospheric conditions and individual receiver sensitivity. One important conclusion from the calibration tests was that Fastloc-GPS errors differed between the two orthogonal dimensions, as has been described for the Argos system [104]. It is therefore advisable to always report the latitude/northing error and longitude/easting error separately.

The on-animal accuracy of Fastloc-GPS loggers may vary somewhat from the accuracy measured during dry tests because of variation in tag placement position on the animal, recording settings, and slowly-changing atmospheric effects such as humidity, pressure, and ionospheric delay. Therefore, in the future, such covariates could be incorporated within SSM frameworks to investigate their relative contributions or to further improve measurement error structures and track accuracy.

This study was motivated by the need for accurate position estimates (with uncertainty) of the whales during relatively short (10–15 min) experimental periods during which naval sonar signals or control stimuli were transmitted under water [28, 73]. In a parallel analysis of the same data set, acoustic propagation modelling will be used for predicting the received sound levels at the locations of the whales. Because relatively short distances between the sonar source and the whales occurred during experimental periods, the estimates of distance and their variability can greatly affect modelled received sound levels. The reconstruction of fine-scale tracks is only the first step in the assessment of humpback whale natural behaviour and responsiveness to sonar; other planned analyses include the classification of discrete behavioural states and behavioural responses based on the reconstructed tracks and auxiliary information. However, visual tracking and Fastloc-GPS are relatively accurate compared to most alternative positioning technologies (such as Argos [62, 78]), and many research questions can be sufficiently addressed without the use of complex methods such SSMs. Possible alternatives are removing part of the data based upon unrealistic speeds [105] or #satellites used in the position calculation [55, 80]. Also, various interpolation methods are available for estimating the track between known position fixes [106].

There are many sources of error that can influence dead-reckoning of animals under water. Eq. 3 hints at one such source of error; the animals naturally move in the water frame of reference and speed is measured in this frame, but the orientation of the whale, used to derive velocity, is measured in the Earth frame (which is eventually of most interest). In addition, water currents may vary with depth due to the Ekman spiral, sensor errors accumulate with time, and speed estimates are often biased and not continuously observed. Also, marine animals do not always move in the same direction as their (flexible) body is oriented due to inertia, buoyancy, and hydrodynamic lift forces (caused by large pectoral fins, for example) [6]. Suction-cup tags can occasionally move over the whale’s body, which means that the correction angles for the conversion from tag to animal frame, as well as the flow noise/speed-relationships, may vary throughout the tag record. Because of this complex mix of errors, we essentially sacrificed some realism for practicality and implemented our relatively simple process model as a correlated random walk on the joint error in horizontal velocity. Visual inspection indicated that ***v***^*cor*^ co-varied with the movement parameters for some animals, but in other tracks small and consistent offsets likely caused by water current appeared to be the dominant factor (Additional file 4: Figures S2-S14). More in-depth analysis of the estimates of ***v***^*cor*^ may provide further insights in the relative contributions of the sources of errors in the tracks.

The model structure presented here was written in the BUGS language (Additional file 1) and is therefore easy to use and adapt. Fitting the models with MCMC had the advantage that the non-Gaussian observation error structures for Fastloc-GPS and bearings were easy to implement, but also made model fitting relatively slow (Table 1). To make model fitting with MCMC possible, measurement errors were not modelled at the time step of the high-resolution data. As a result, the model underestimated the positional uncertainty in the track when fixes were not observed. This effect was likely to be small for the short track segments in this study but will increase with the time since the most recent location measurement. More realistic confidence bounds could conceivably be added to the track segments between surfacings using a Kalman filter that is conditioned on the start and end points of each track realisation.

By accounting for the observation errors in the position fixes, our model can provide a clear improvement over simpler methods to georeference dead-reckoning tracks [5]. Similarly, compared to tracks derived only from position fixes [73], the inclusion of dead-reckoning data greatly improved the level of detail in the reconstructed humpback whale tracks (Additional file 4: Figures S2-S14). Cross-validation analyses confirmed that out-of-set Fastloc-GPS locations were better predicted by our model framework than by simpler track reconstruction methods that do not allow for positional observation error. Independent validation of our technique might be (partially) possible in the future using double tagging experiments (e.g. [56]) with conventional GPS, using passive acoustic locations of animals that vocalise underwater [107], or using current velocity data from acoustic Doppler current profilers or numerical ocean models.

Being a recursive method, dead-reckoning generally results in positional errors that increase with time, and the speed of the water current may have a particularly large influence on these errors. Knowing the rate at which model performance deteriorated would be useful for scientists studying different species or for users of animal data loggers who need to decide on position sampling schemes. However, a preliminary analysis (not shown here) of the cross-validation errors against time to the nearest Fastloc-GPS position did not consistently demonstrate this trend of decreasing model performance, likely because of the relatively large contribution of Fastloc-GPS observation errors and because time intervals between locations were relatively short (<10 min).

The integration of Fastloc-GPS, depth, speed and inertial sensor data is an exciting development that opens the door to the reconstruction of georeferenced 3-dimensional movement tracks with relatively high precision compared to existing positioning methods. As similar track reconstruction approaches are currently being developed [107, 108], a systematic comparison of the tracks produced by the different techniques in the future would be valuable. High-resolution animal tracks have the potential to answer fascinating scientific questions about, for example, predator movements in relation to prey fields, dynamics of group movement, impacts of human disturbance on behaviour, and how foraging effort and success relate to individual and population fitness. The advancement of bio-logging technology is rapid and, in our opinion, scientists will benefit from the use and development of analysis methods that make the most out of the growing wealth of information.

## Availability of supporting data

The data set of whale 11, the JAGS model code, and examples of MATLAB code used in the analysis are included as additional files with the article (Additional file 1).

## Additional files

Additional file 1:
**Example data set, JAGS model, and MATLAB code.** Compressed file including the data set of whale 11, the JAGS model code, and example MATLAB code to process the data, fit the model, and reconstruct the high-resolution track shown in Fig. 3. (RAR 2041 kb) Additional file 2: Figure S1. Boxplots of the Fastloc-GPS positional errors for the three data loggers (29409, 29 402, and 29 520, from top to bottom), four calibration test sites (A: 56.33°N, 2.78°W; B: 69.68°N, 18.99°E; C: 78.24°N, 15.54°E; D: 64.92°N, 23.25°W), and six #satellite bins (4, 5, 6, 7, 8, and >8). The sample size for each subset is indicated on the right vertical axis. Outlier data points were omitted to improve readability. (PDF 19 kb) Additional file 3: Table S1. Posterior statistics and $\widehat{R}$ values. Table with the mean, SD, and 95 % CI for the posterior distributions of parameters *σ*_*x*_, *σ*_*y*_, *v*_*x*,1_^*cor*^, *v*_*y*,1_^*cor*^, *x*_*x*,1_, *x*_*y*,1_, and the values of convergence statistic $\widehat{R}$. (XLSX 17 kb) Additional file 4: Figures S2-S14. Figures of the reconstructed tracks and movement parameter time series for all whales. See the caption of Fig. 3 for more details about the information that is plotted. Note that the scale of the depth axis differs per whale. (PDF 18518 kb) Additional file 5: Figure S15. Posterior distributions for all whales. (PDF 91 kb) Additional file 6: Figure S16. One-dimensional Fastloc-GPS errors. Positional errors during calibrations were represented as radial distances from the median and plotted against the cumulative percentage of positions for comparison with other studies. Each line represents a subset of data based upon the number of satellites (4 to 12) used for the position calculation. The insert shows the pdfs for the 9 satellite coverage categories. The graphs were truncated at 100 m for clarity. (PDF 12 kb)

## Abbreviations

BUGS: Bayesian inference Using Gibbs Sampling
EGNOS: European geostationary navigation overlay service
GPS: Global positioning system
JAGS: Just another Gibbs sampler
LRF: Laser range finder
MCMC: Markov chain Monte Carlo
SSM: State space model
UTC: Coordinated universal time
UTM: Universal transverse mercator
VHF: Very high frequency
