# Supplementary figures and images for: A path reconstruction method integrating dead-reckoning and position fixes applied to humpback whales
Source: Mov Ecol. 2015 Sep 21;3:31. doi: 10.1186/s40462-015-0061-6 (PMC4576411; doi:10.1186/s40462-015-0061-6)

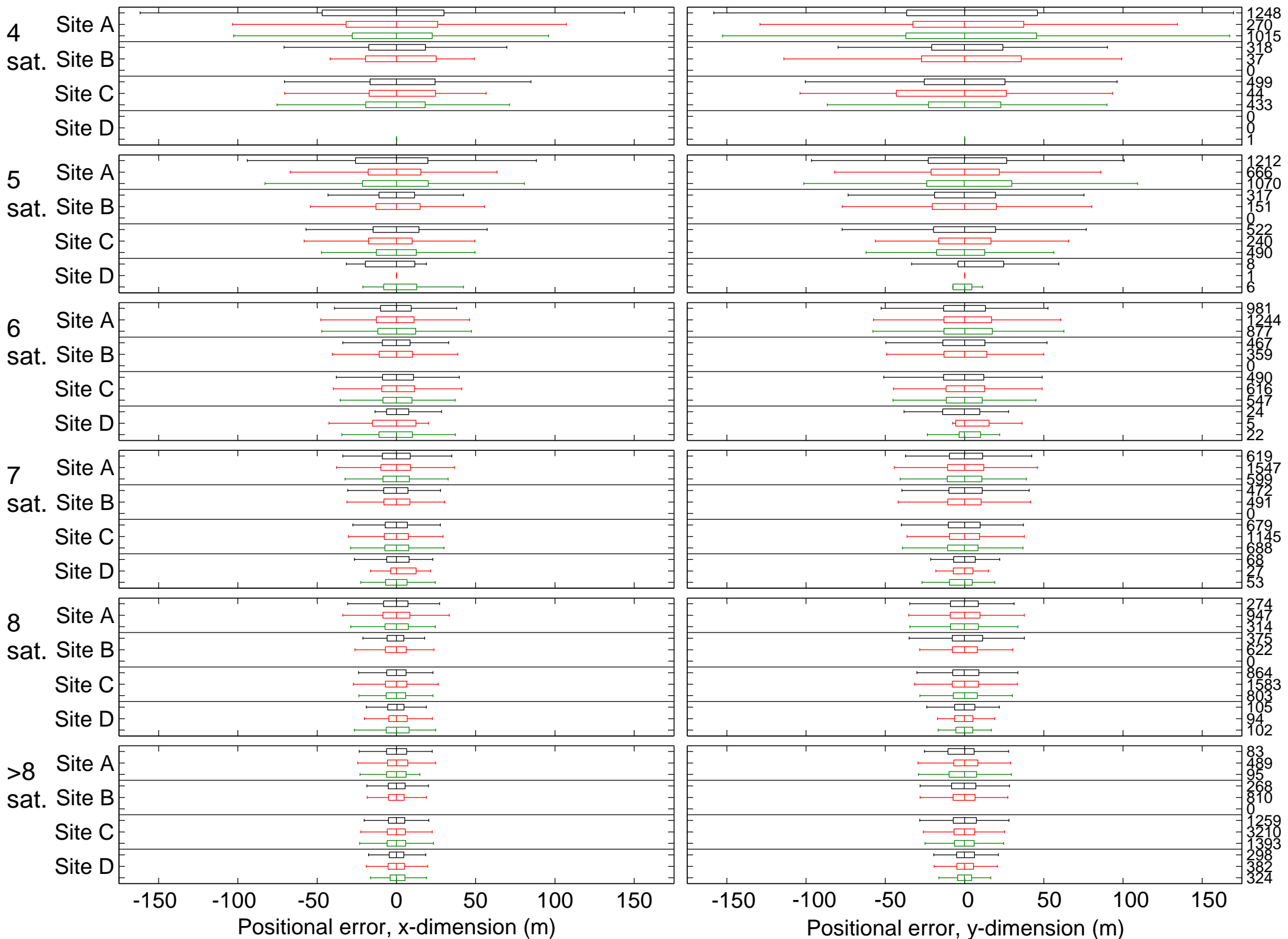

Supplement: Additional file 2: Figure S1. — Boxplots of the Fastloc-GPS positional errors for the three data loggers (29409, 29 402, and 29 520, from top to bottom), four calibration test sites (A: 56.33°N, 2.78°W; B: 69.68°N, 18.99°E; C: 78.24°N, 15.54°E; D: 64.92°N, 23.25°W), and six #satellite bins (4, 5, 6, 7, 8, and >8). The sample size for each subset is indicated on the right vertical axis. Outlier data points were omitted to improve readability. (PDF 19 kb) [file 40462_2015_61_MOESM2_ESM.pdf]

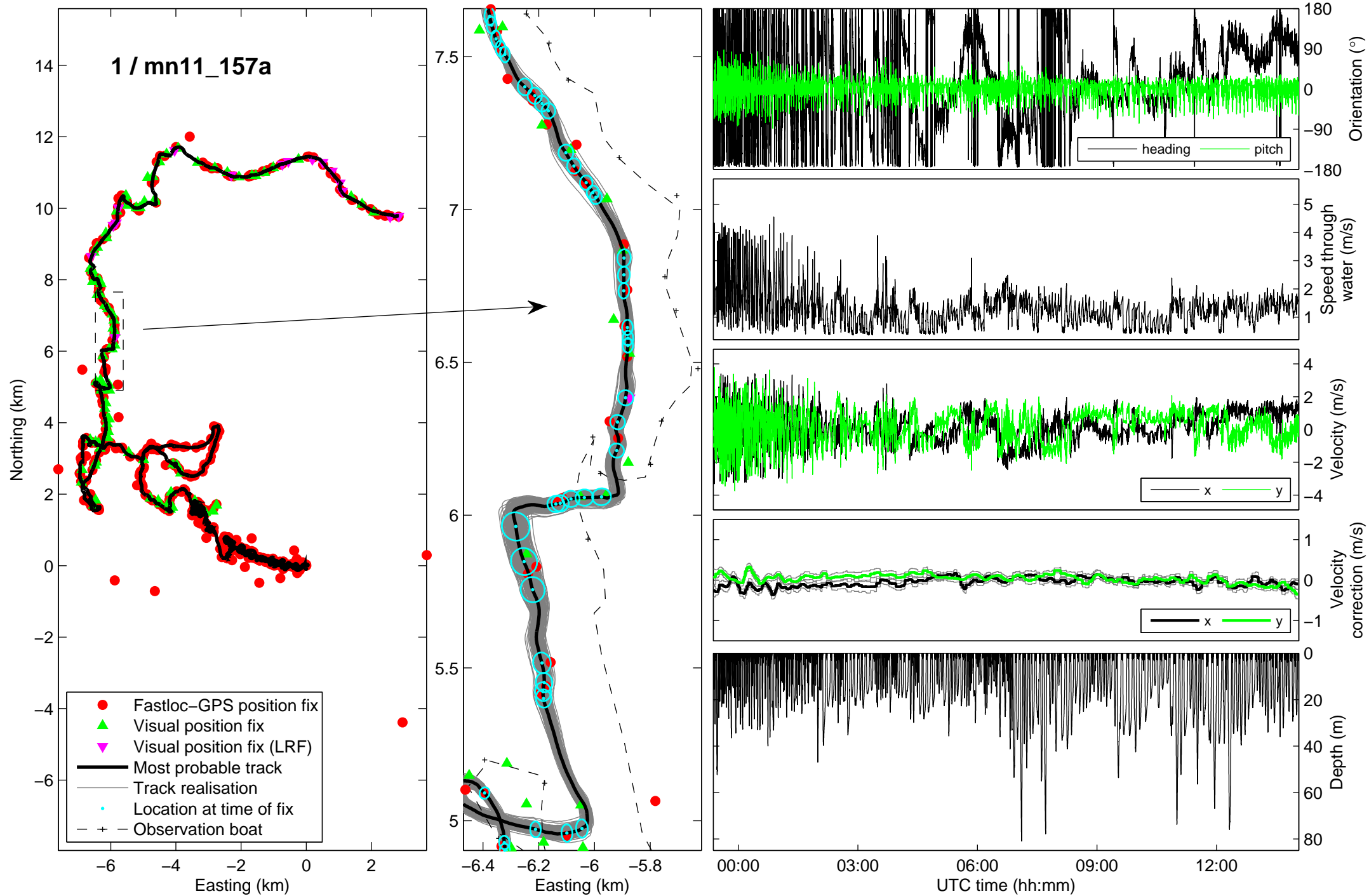

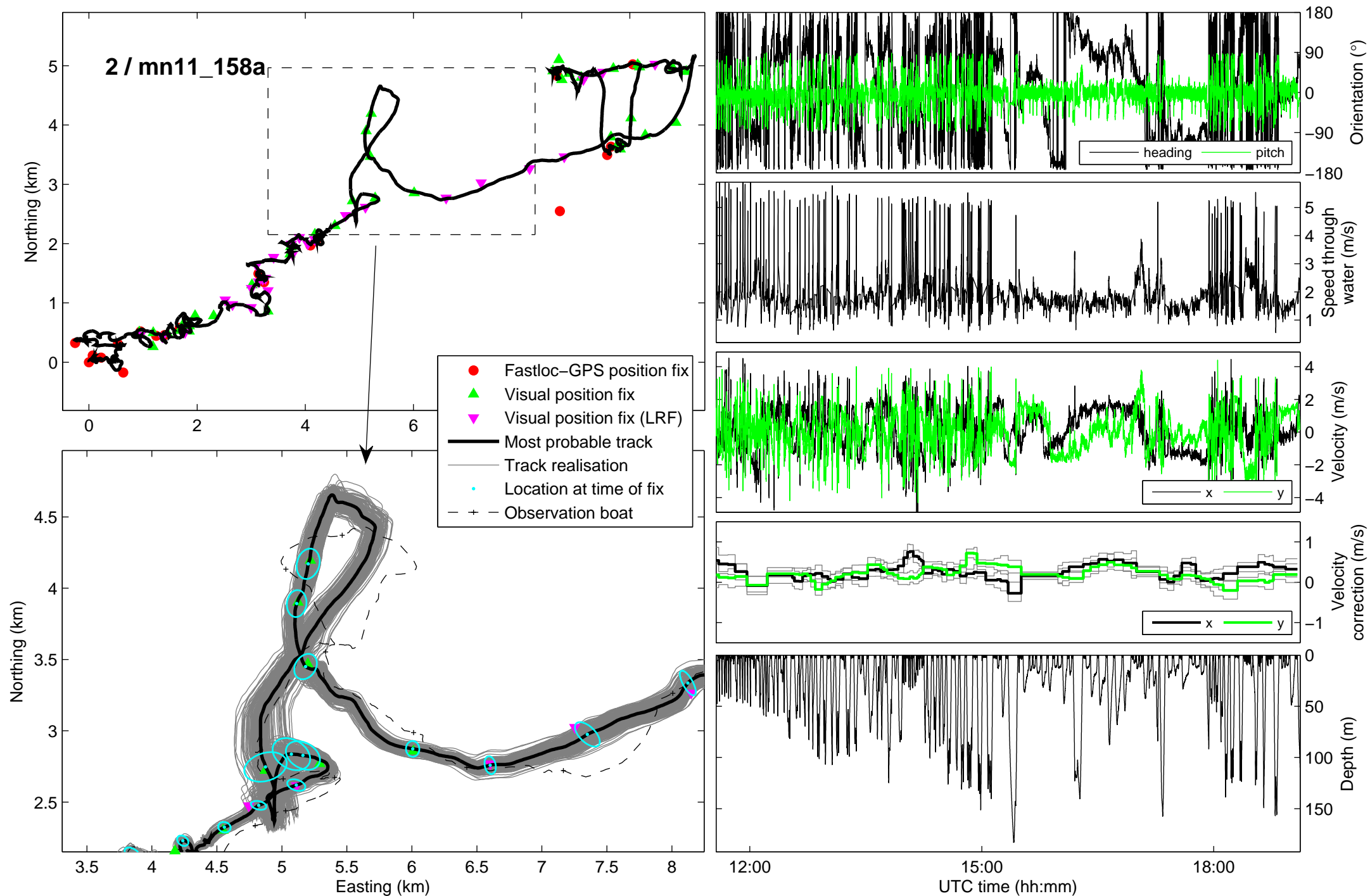

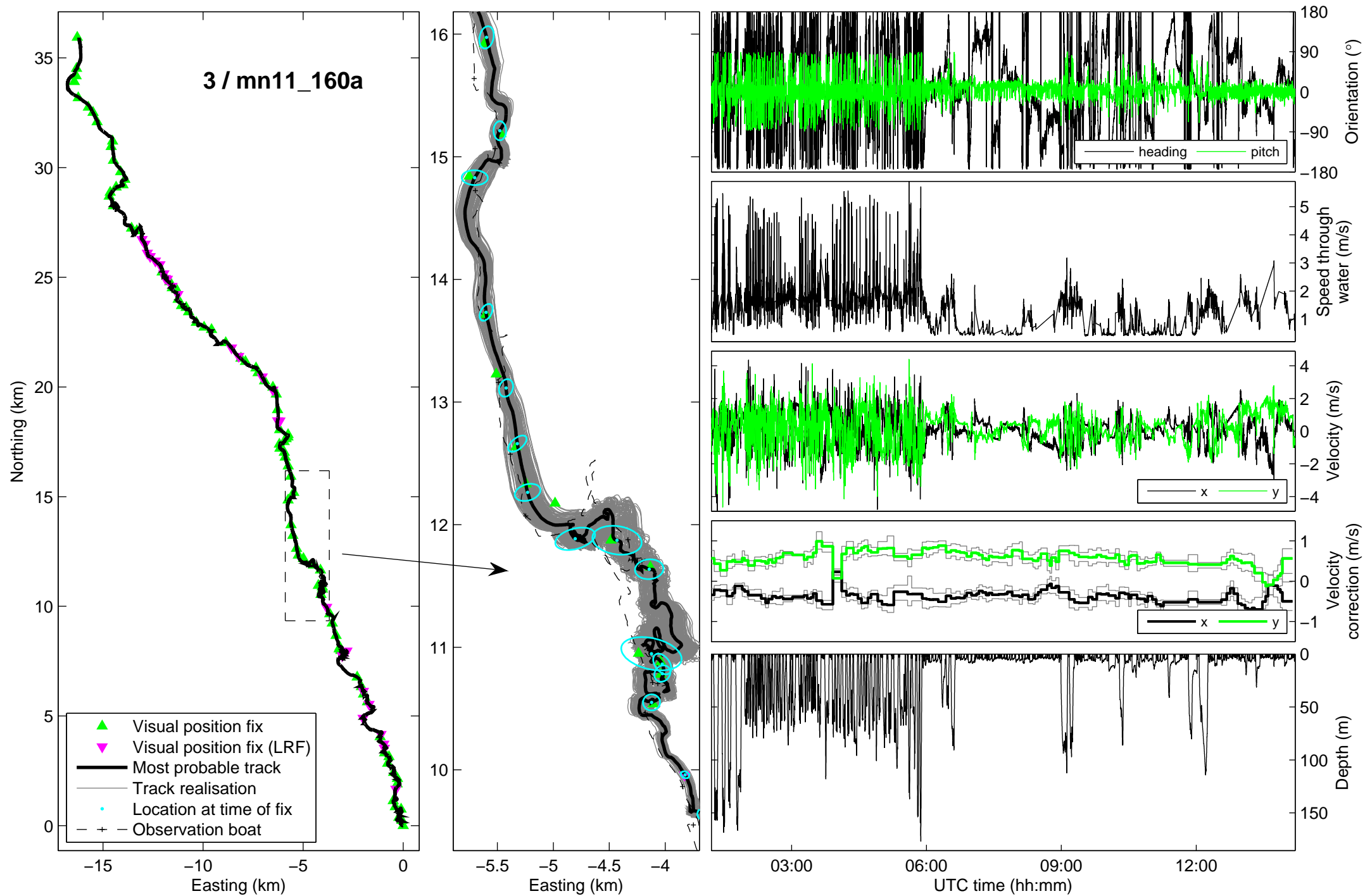

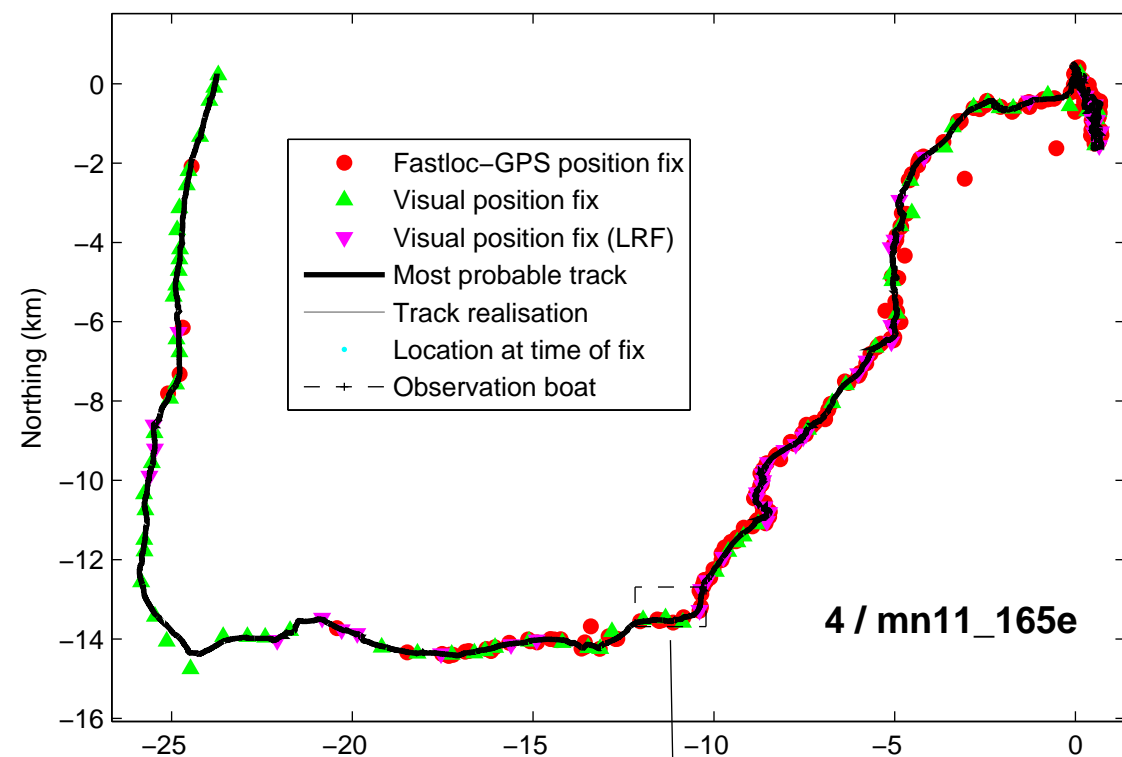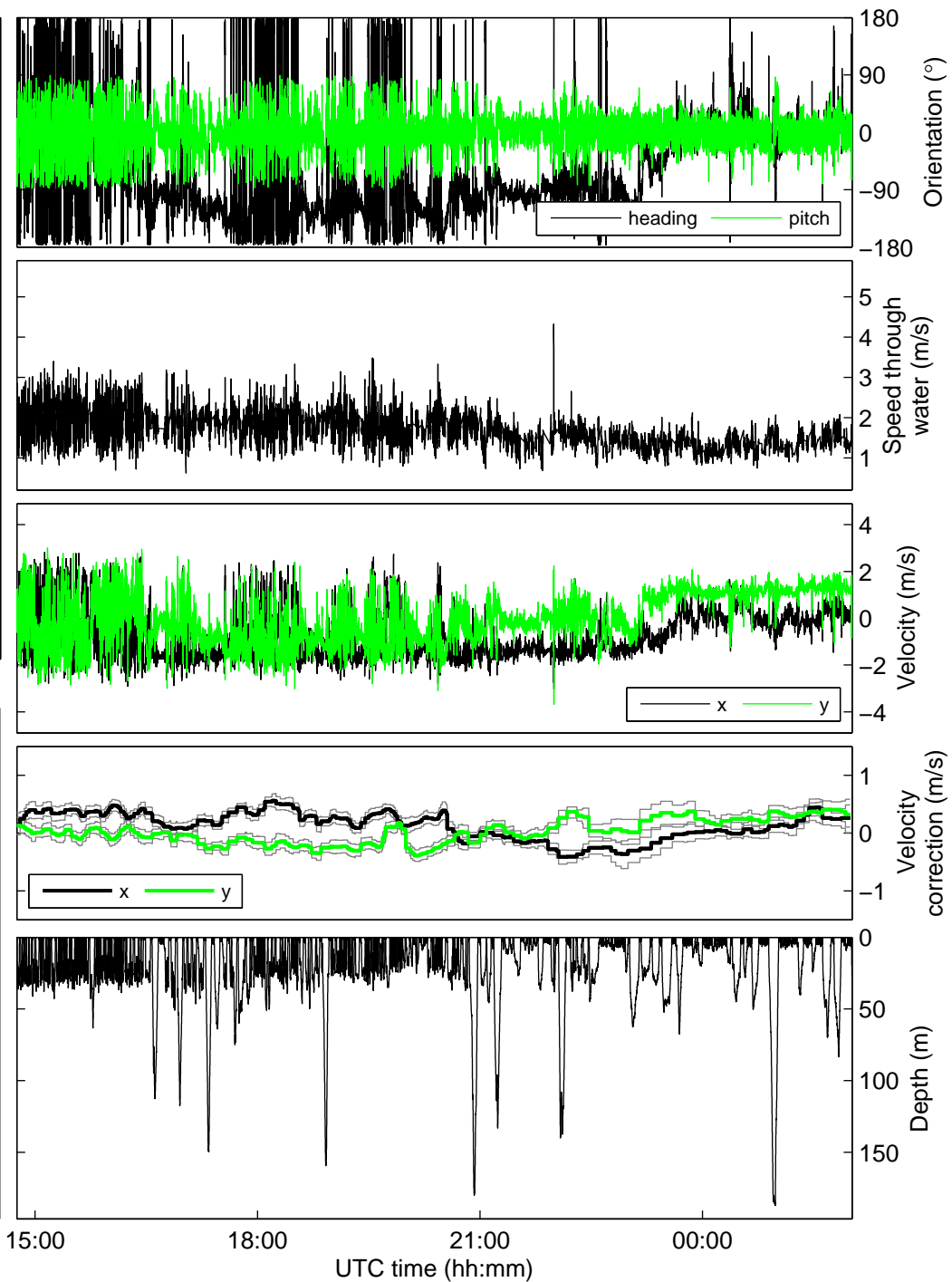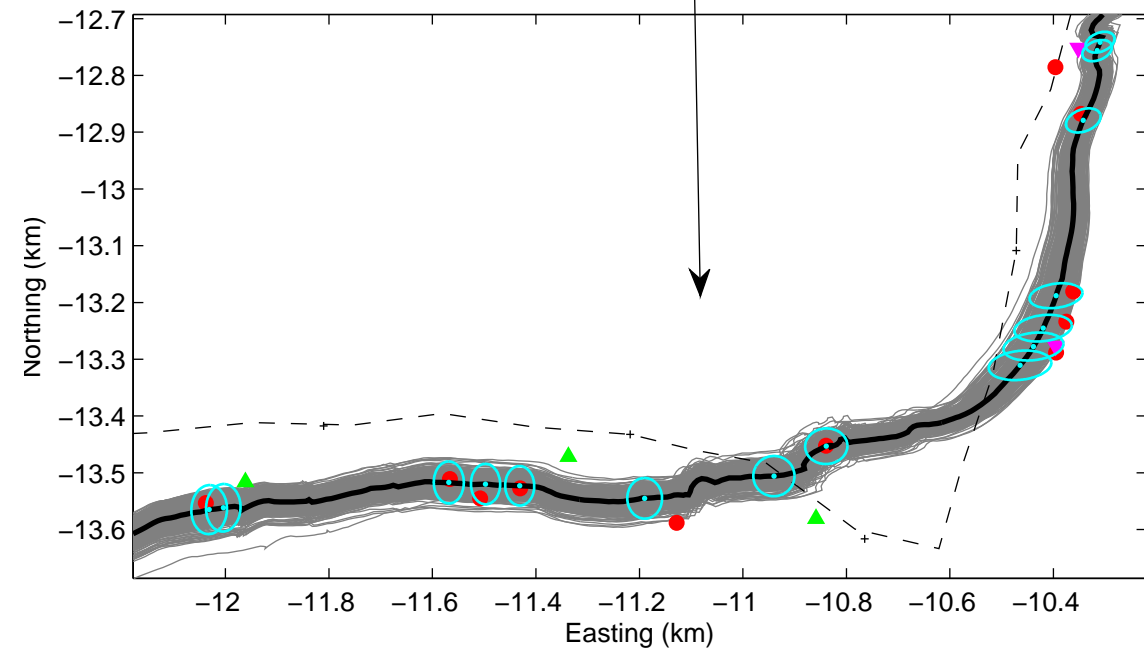

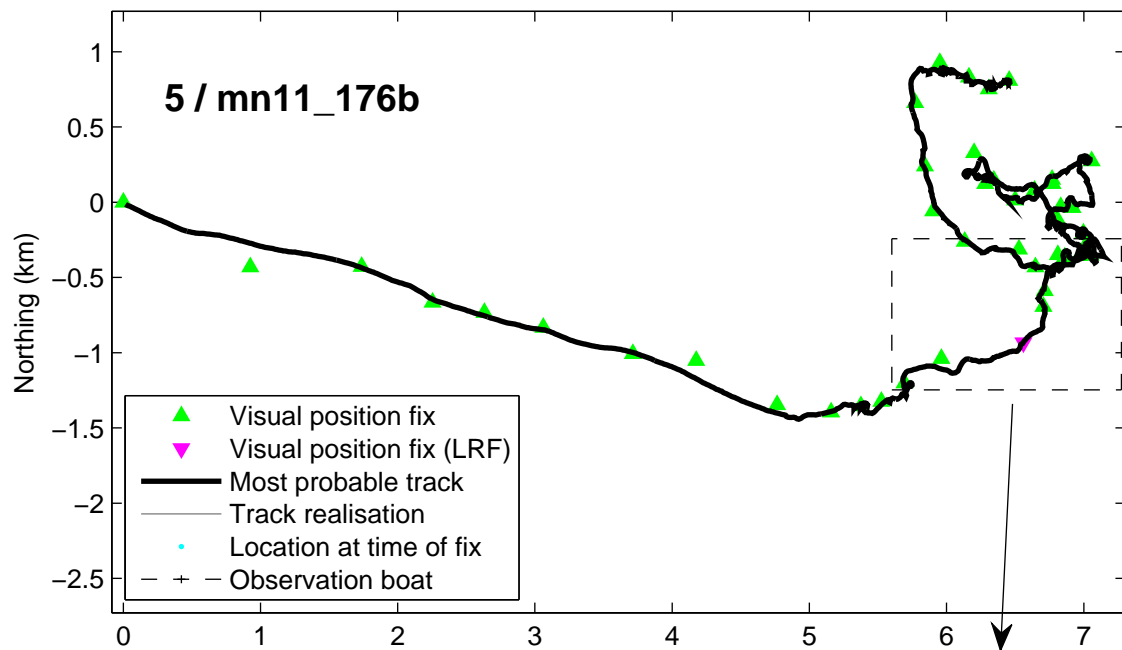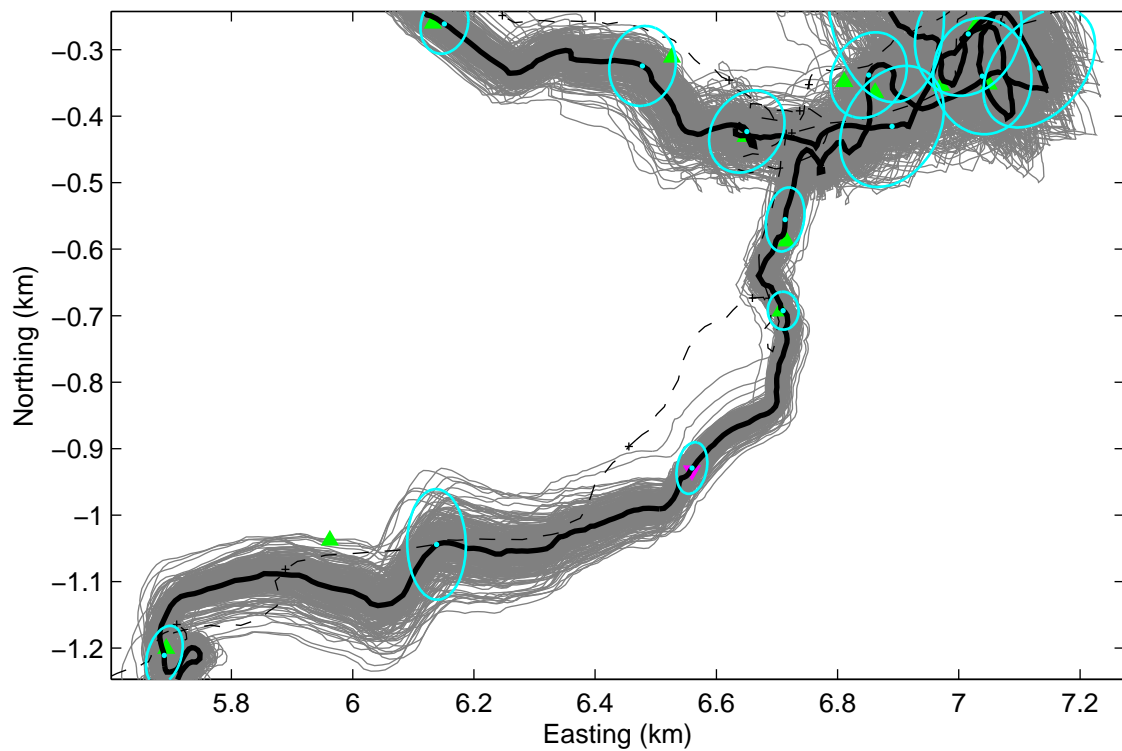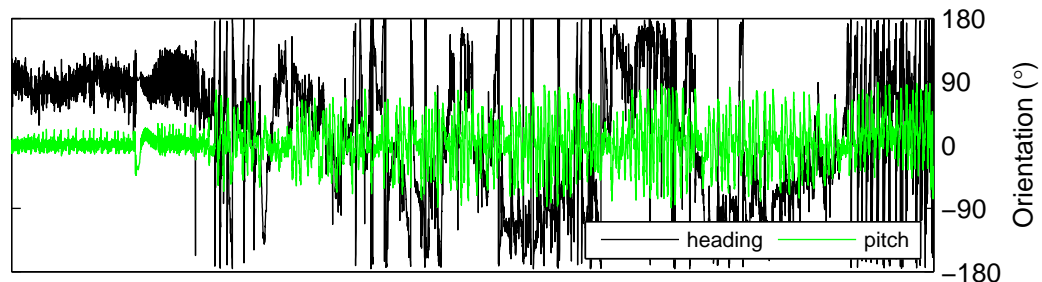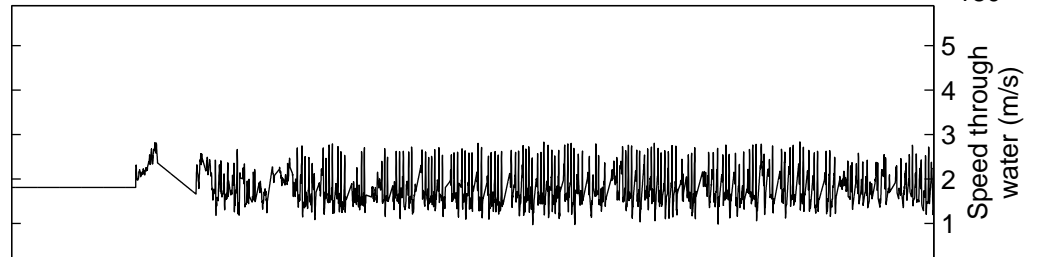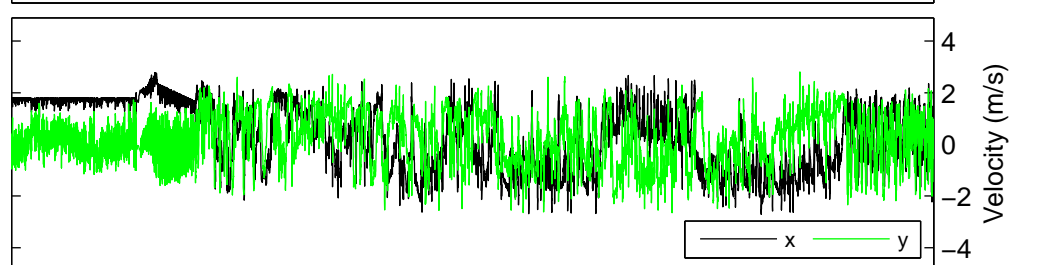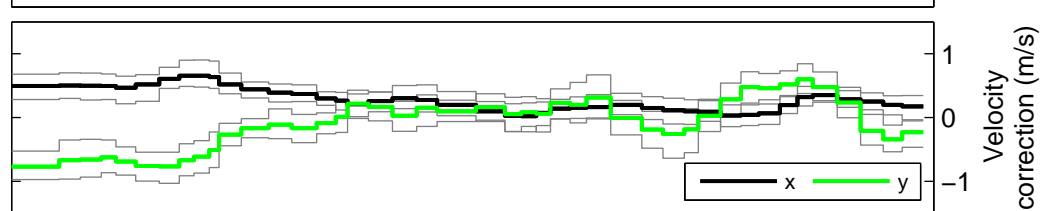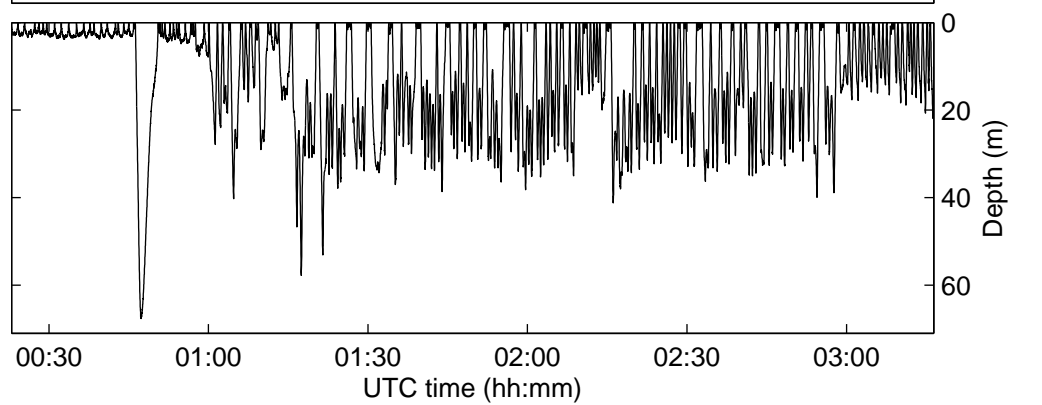

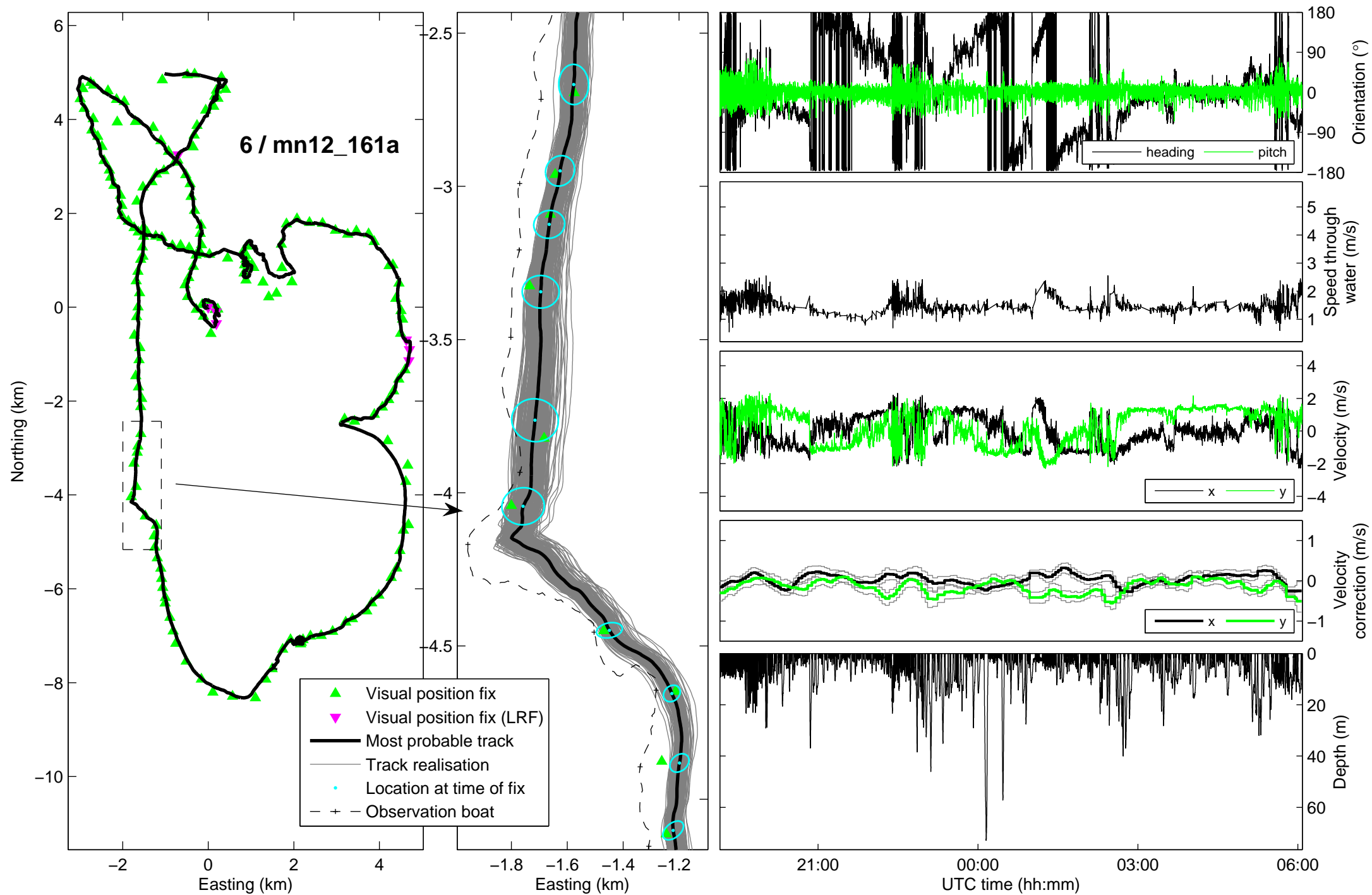

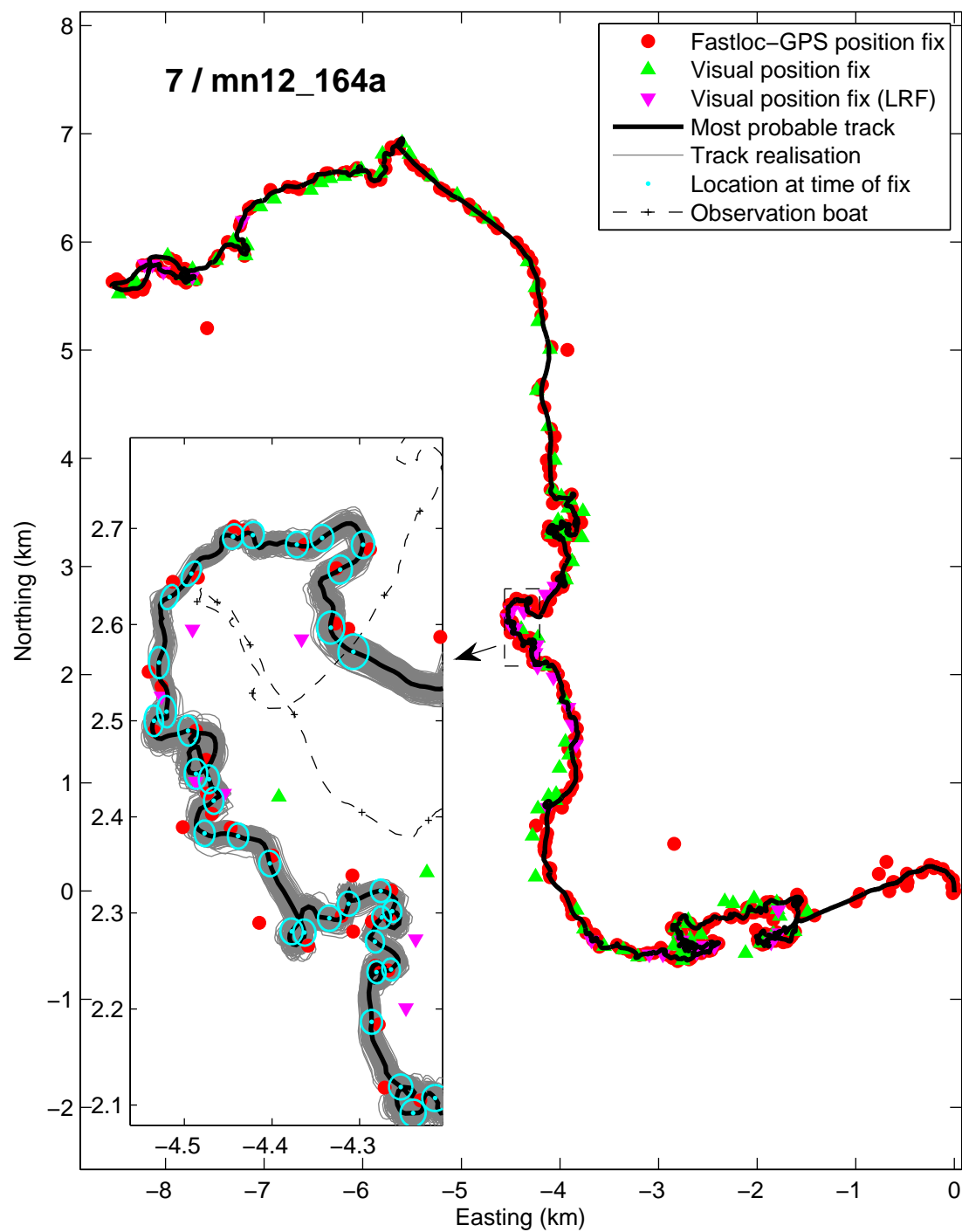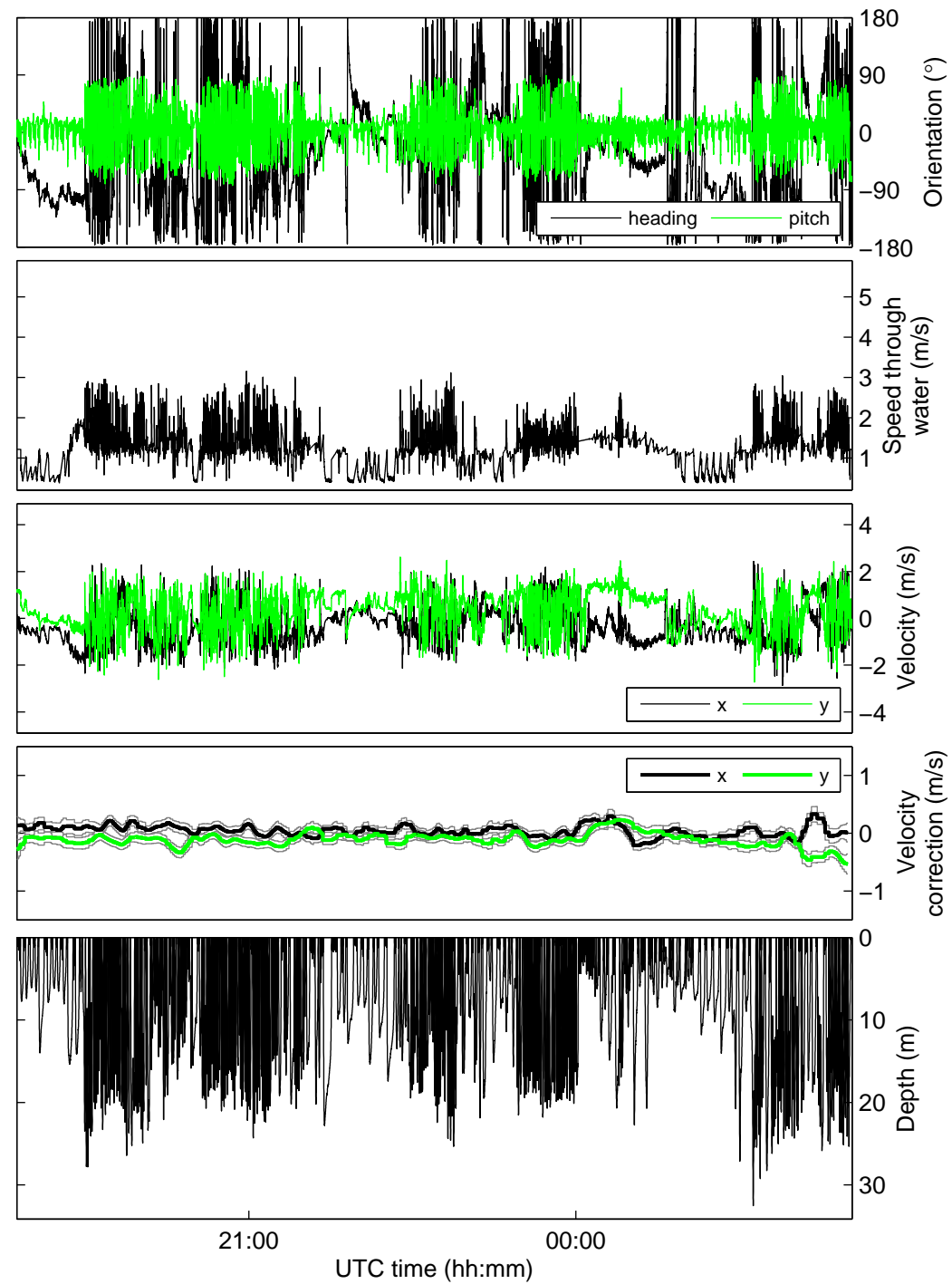

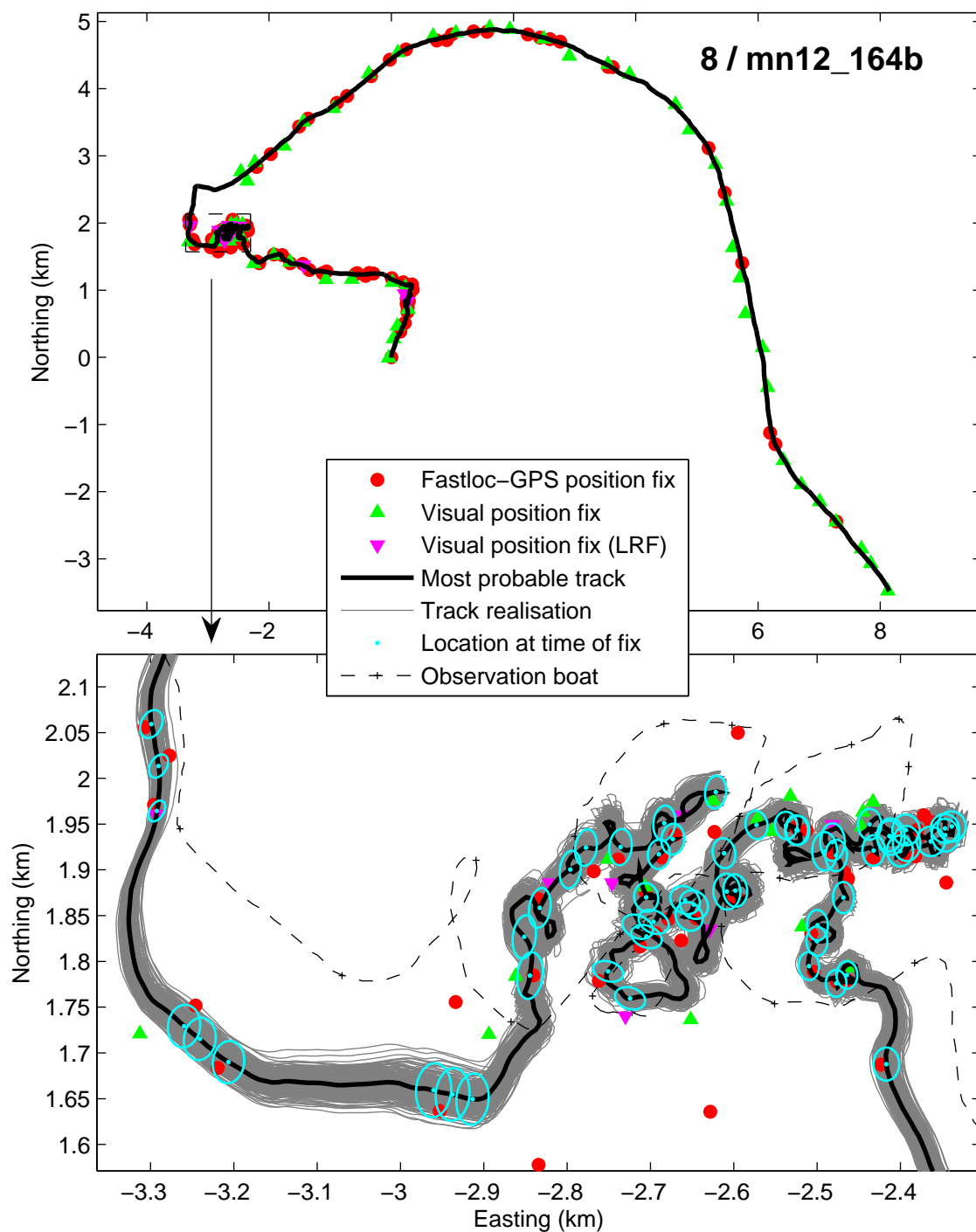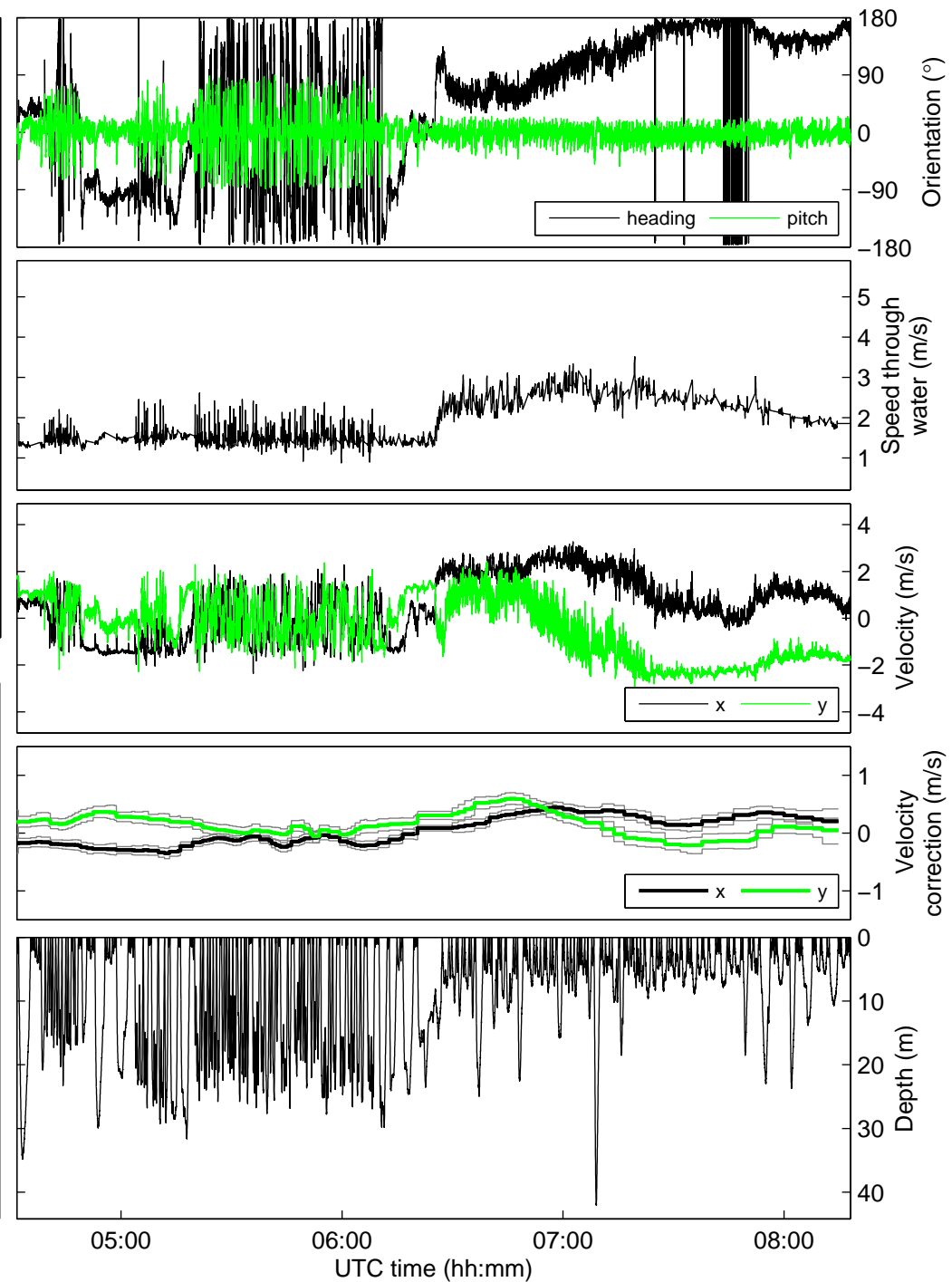

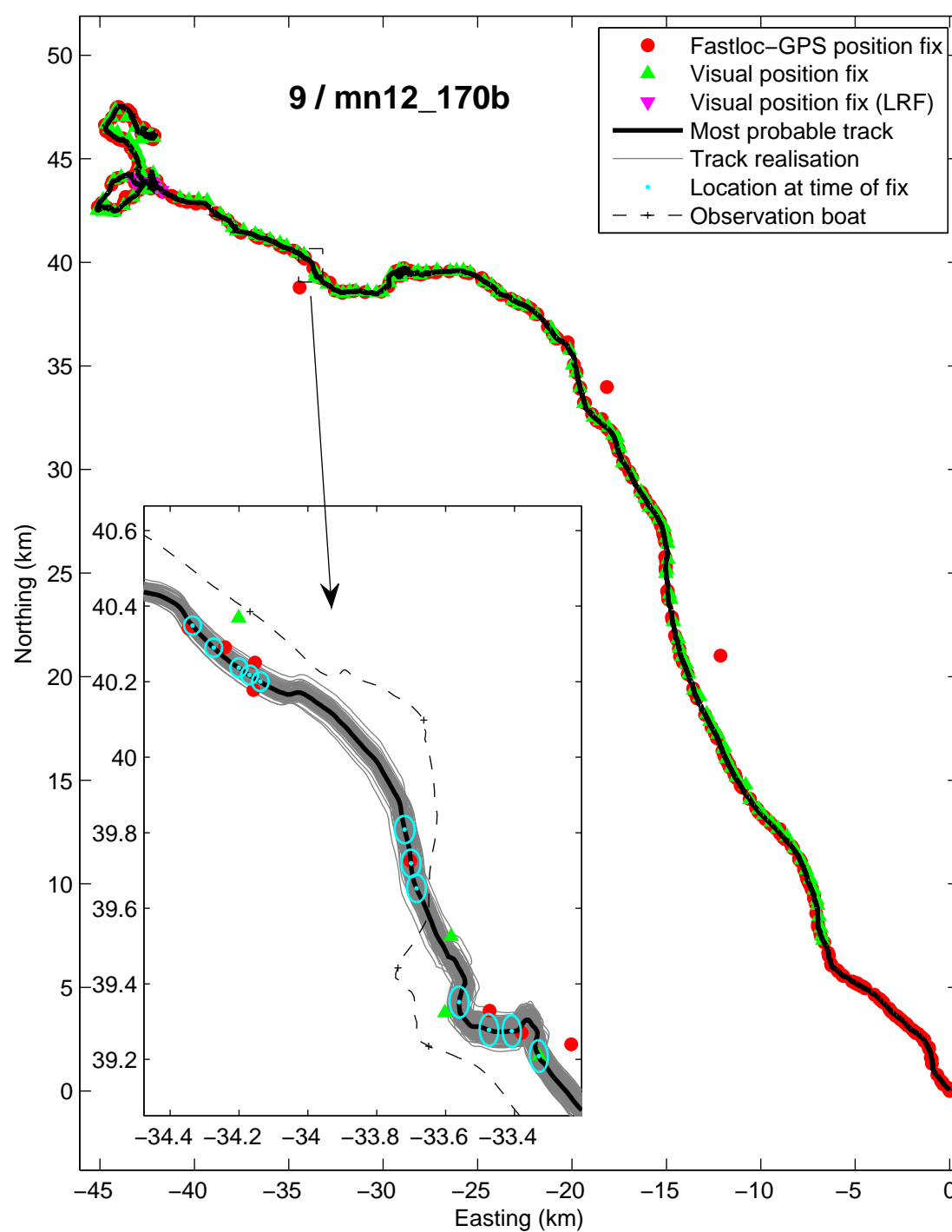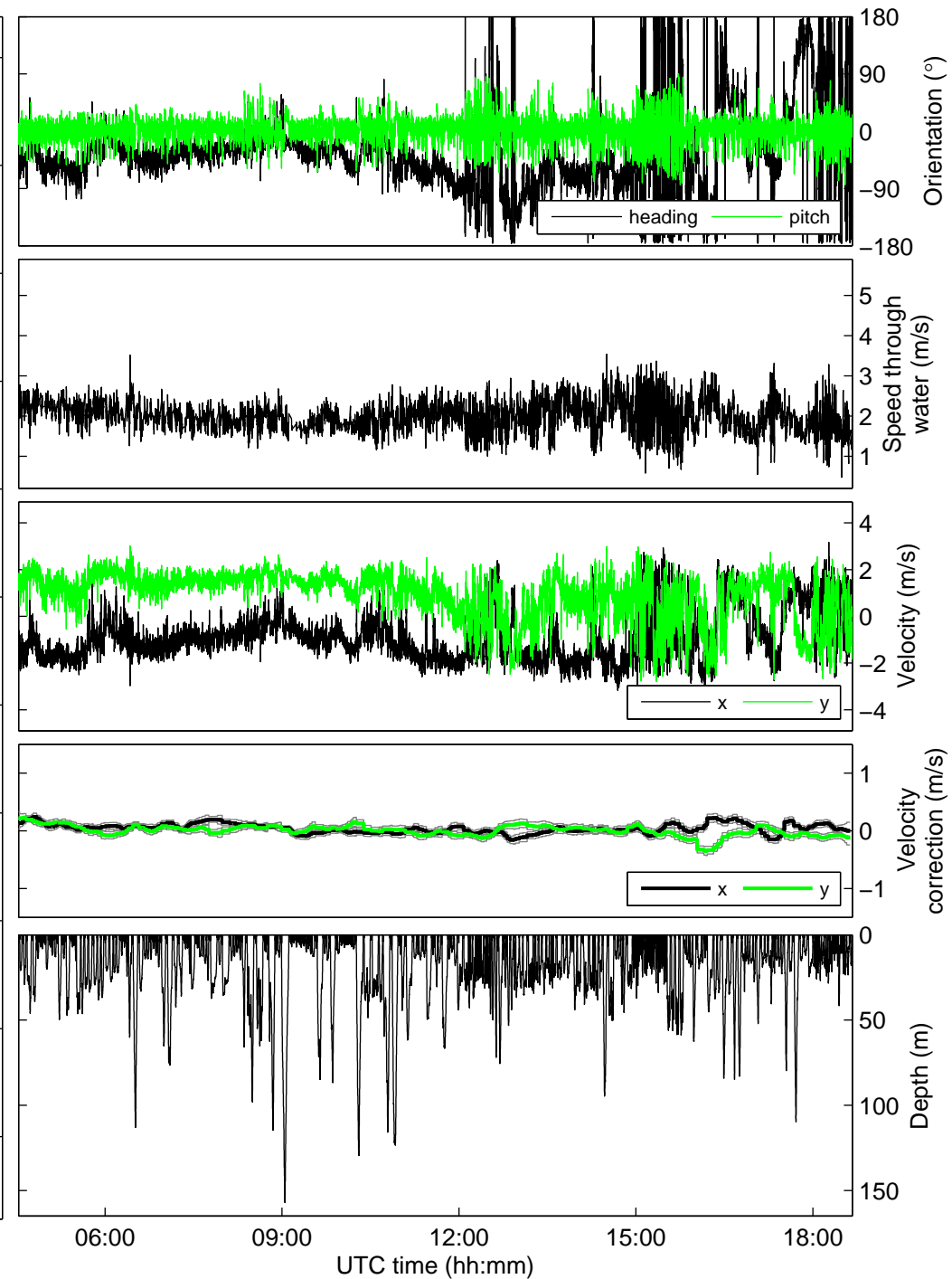

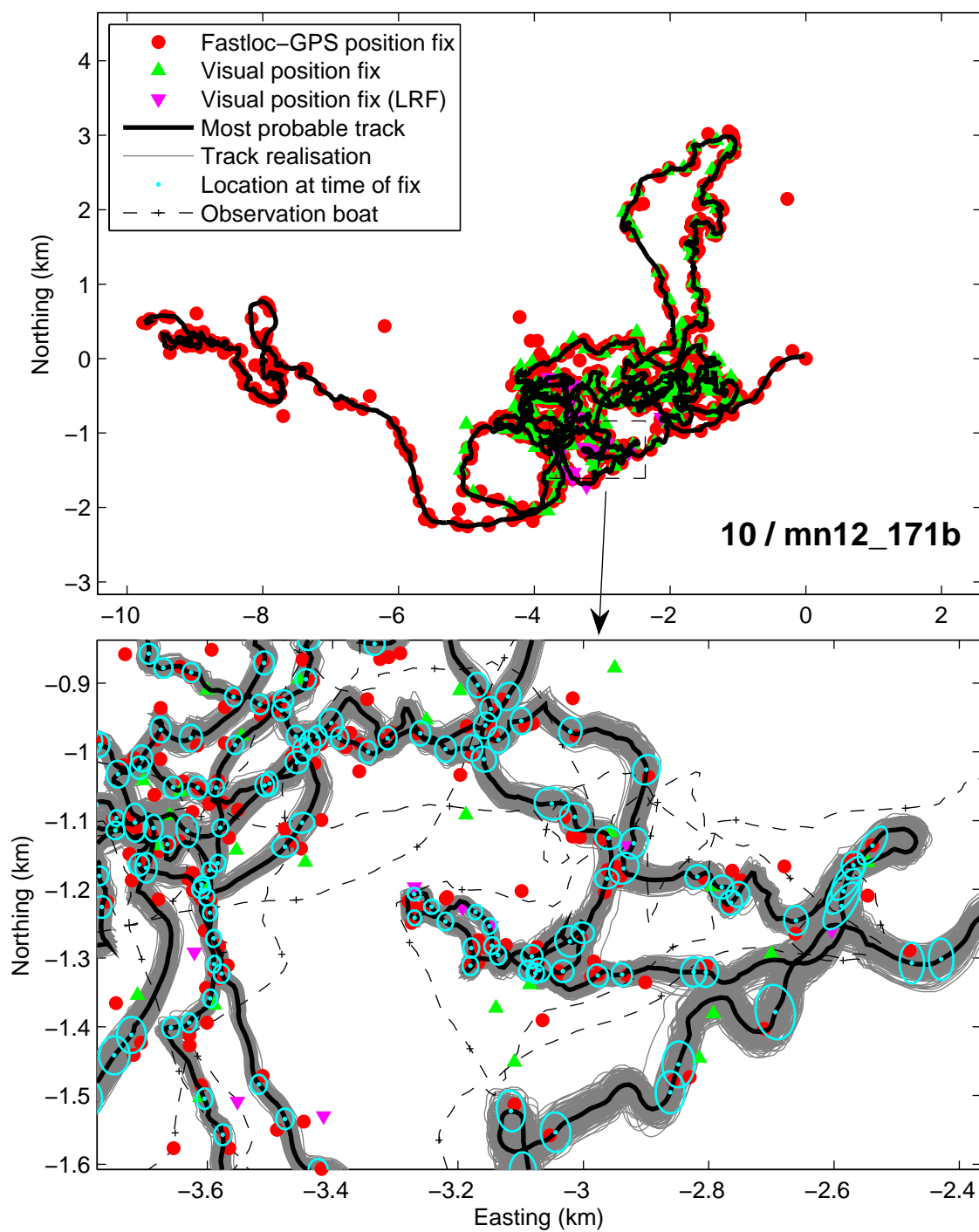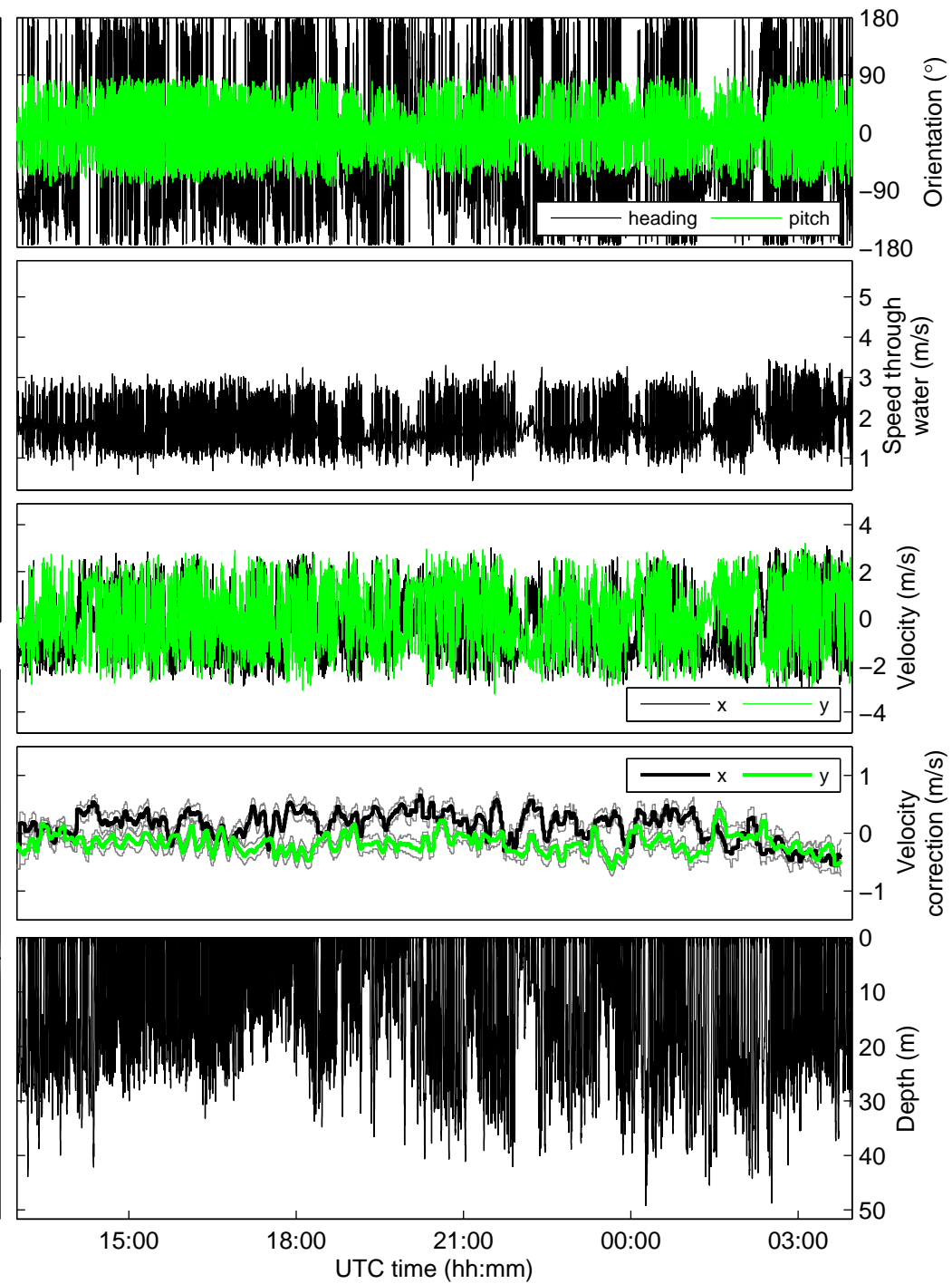

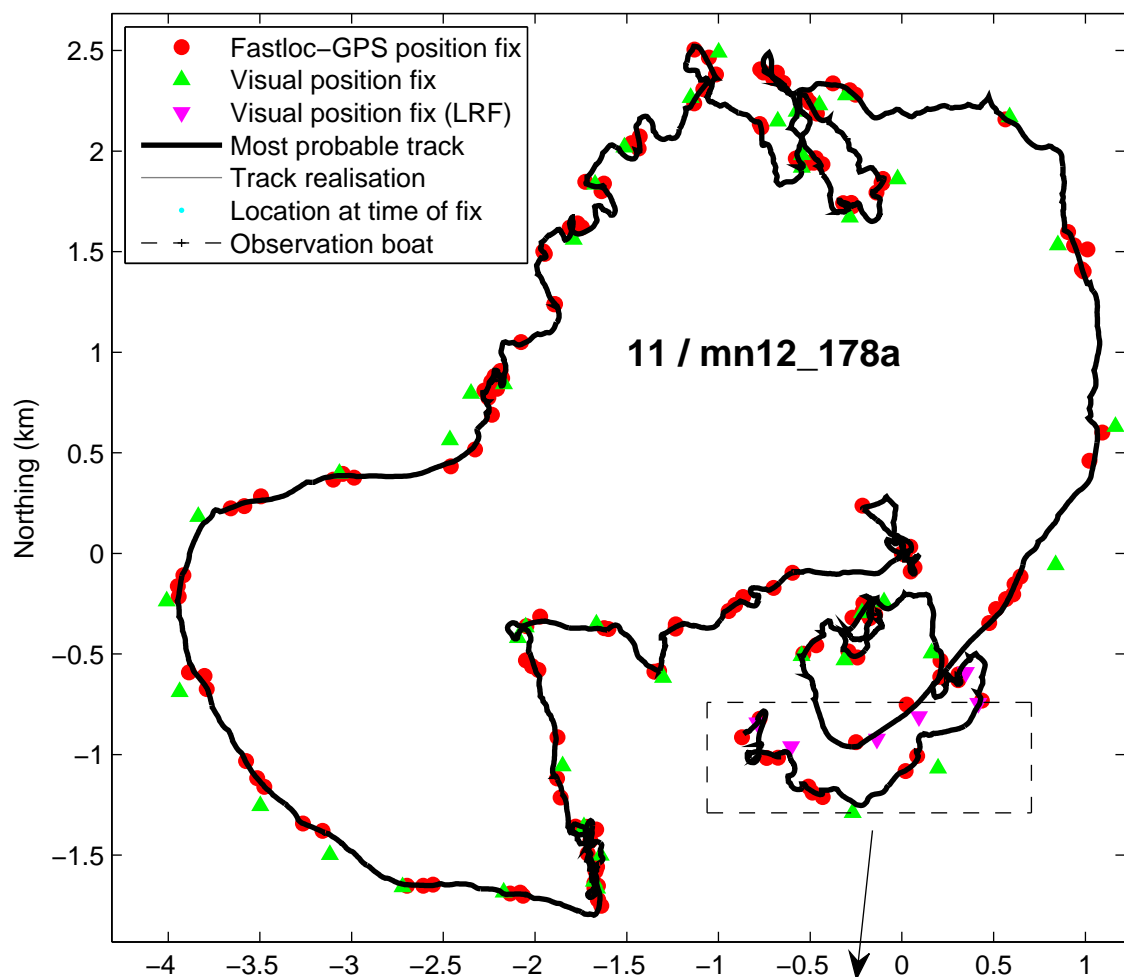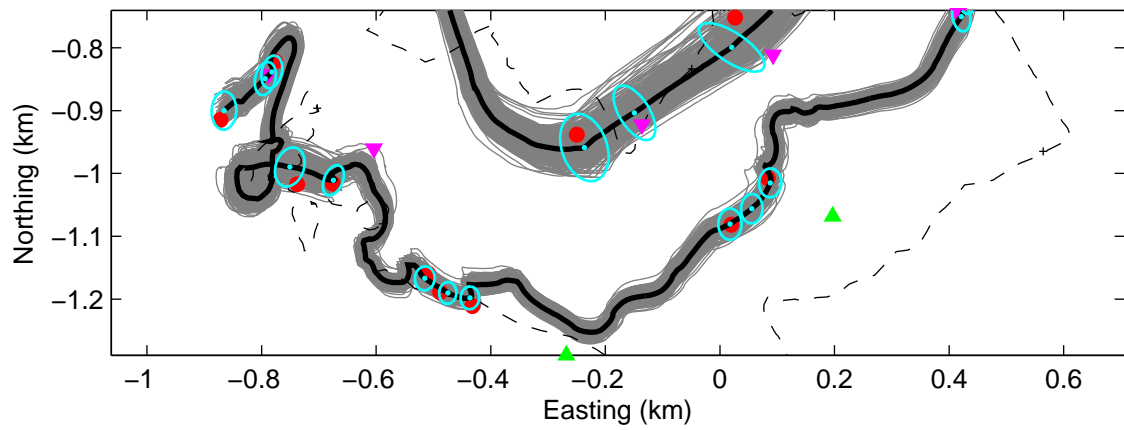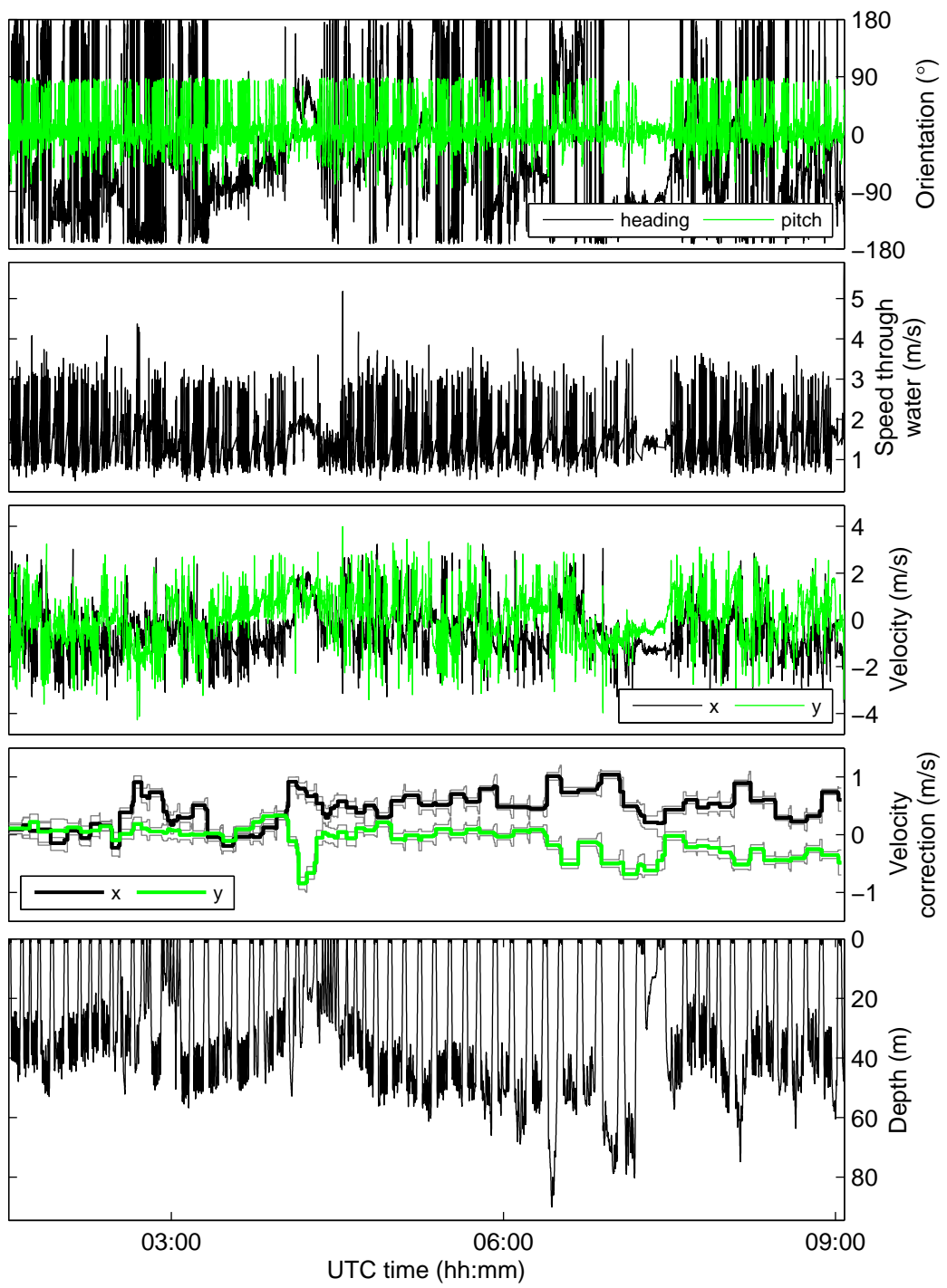

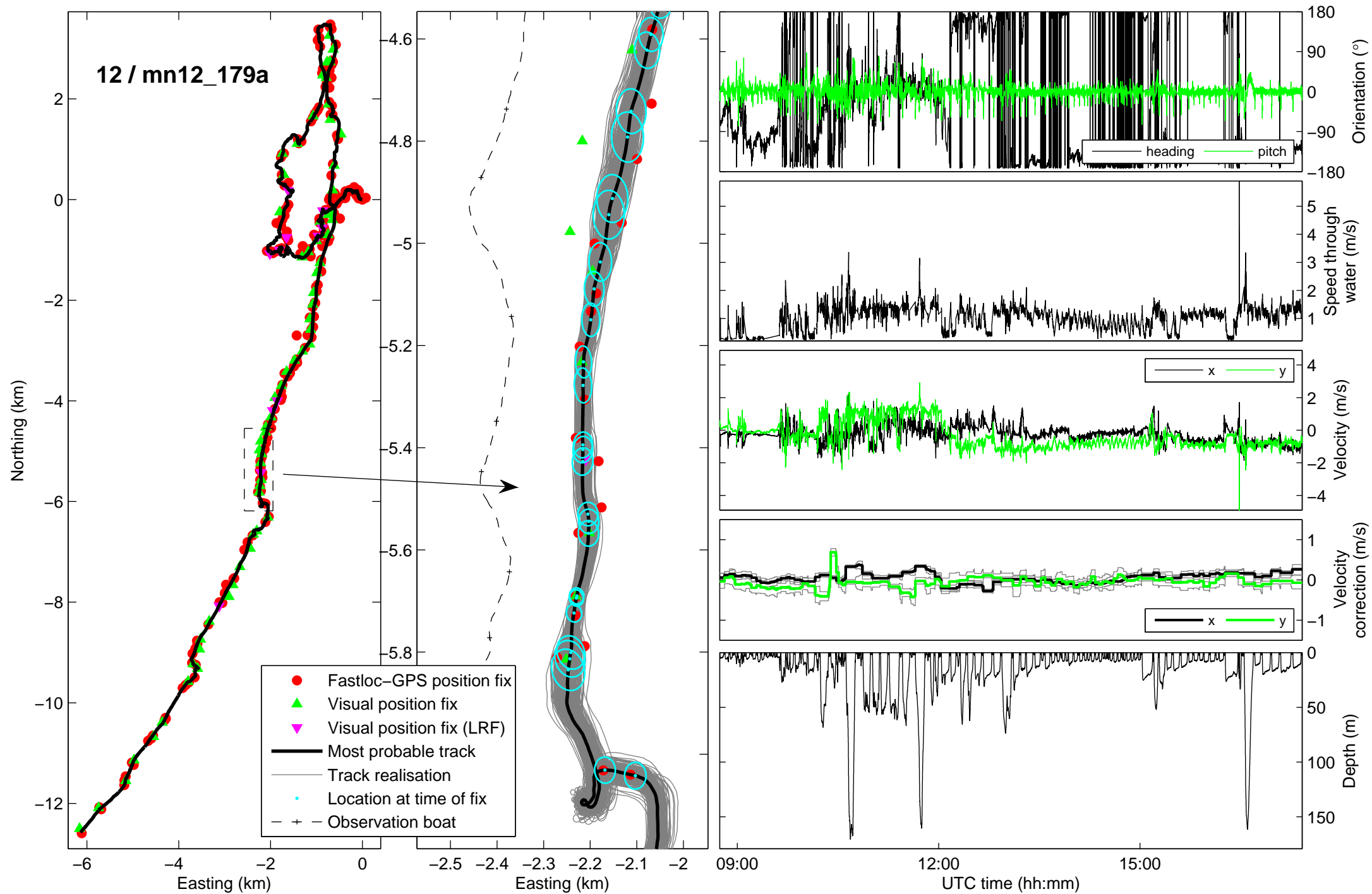

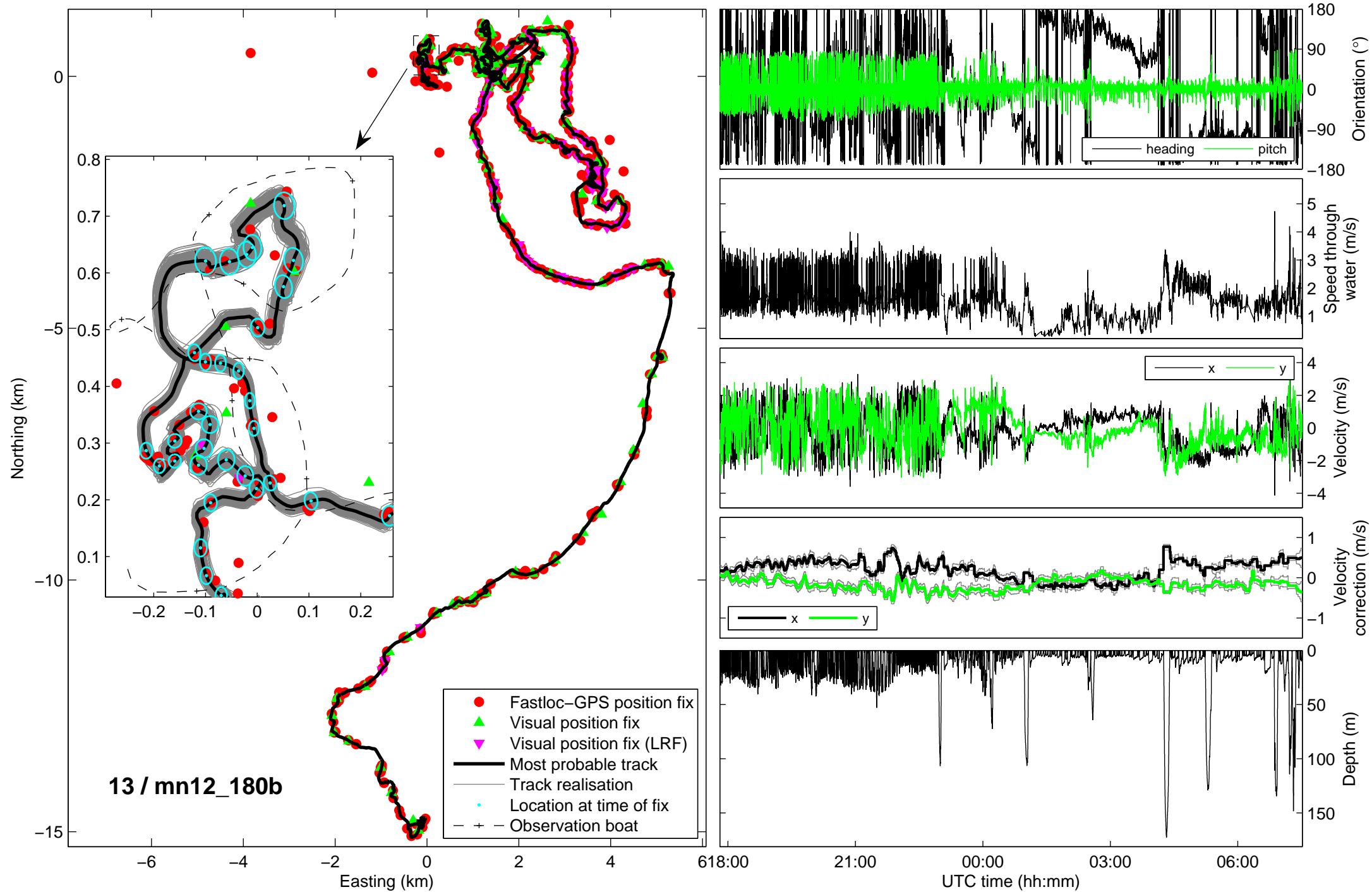

Supplement: Additional file 4: Figures S2-S14. — Figures of the reconstructed tracks and movement parameter time series for all whales. See the caption of Fig. 3 for more details about the information that is plotted. Note that the scale of the depth axis differs per whale. (PDF 18518 kb) [file 40462_2015_61_MOESM4_ESM.pdf]

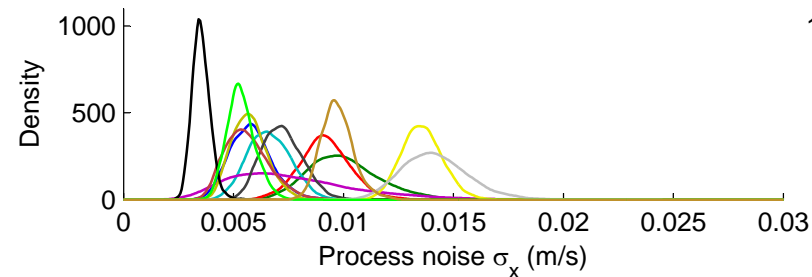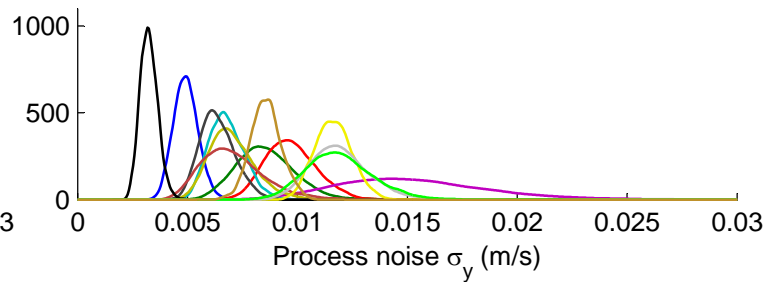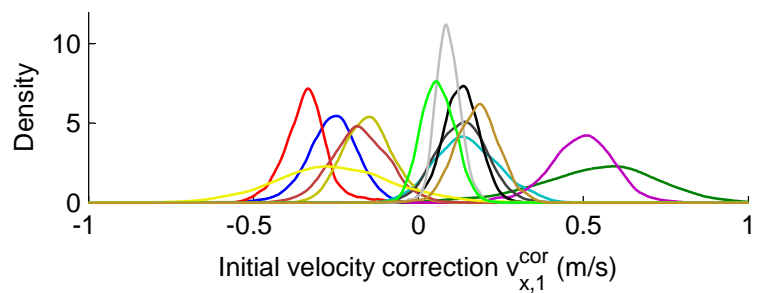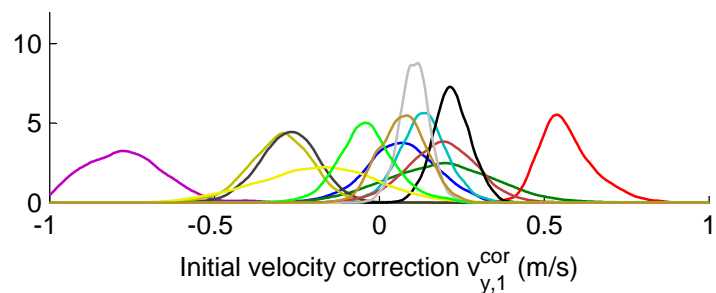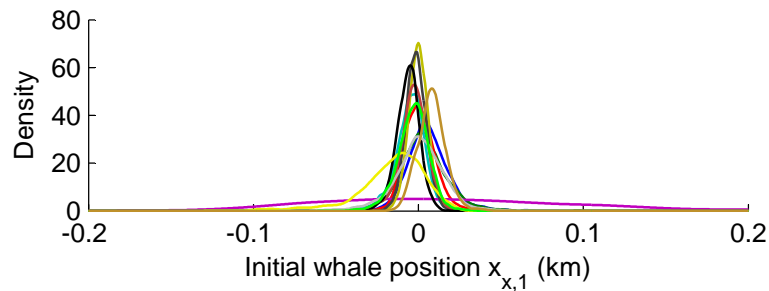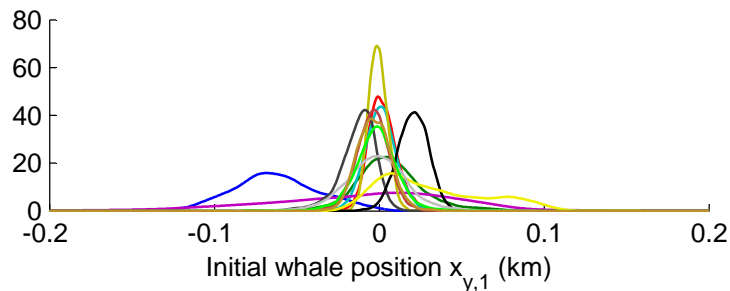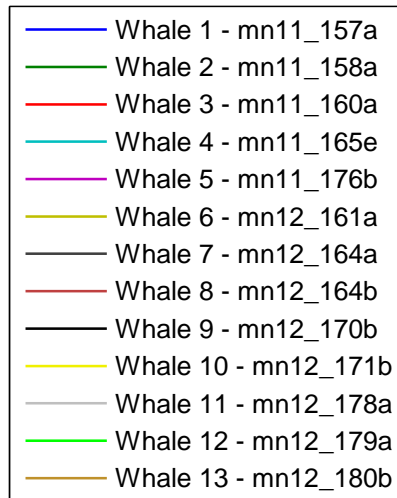

Supplement: Additional file 5: Figure S15. — Posterior distributions for all whales. (PDF 91 kb) [file 40462_2015_61_MOESM5_ESM.pdf]

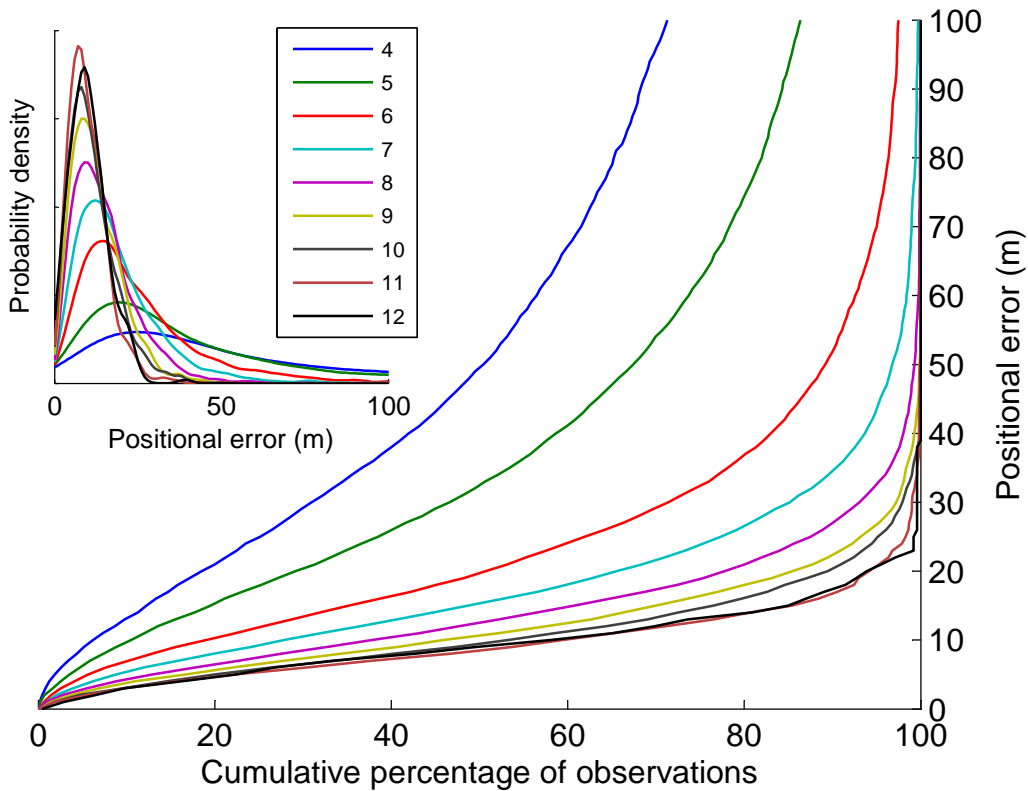

Supplement: Additional file 6: Figure S16. — One-dimensional Fastloc-GPS errors. Positional errors during calibrations were represented as radial distances from the median and plotted against the cumulative percentage of positions for comparison with other studies. Each line represents a subset of data based upon the number of satellites (4 to 12) used for the position calculation. The insert shows the pdfs for the 9 satellite coverage categories. The graphs were truncated at 100 m for clarity. (PDF 12 kb) [file 40462_2015_61_MOESM6_ESM.pdf]
